# The clinical relevance of serum versus CSF NMDAR autoantibodies associated exclusively with psychiatric features: a systematic review and meta-analysis of individual patient data

**DOI:** 10.1007/s00415-022-11224-6

**Published:** 2022-07-05

**Authors:** Graham Blackman, Mao Fong Lim, Thomas Pollak, Adam Al-Diwani, Mkael Symmonds, Asif Mazumder, Ben Carter, Sarosh Irani, Anthony David

**Affiliations:** 1 Department of Psychosis Studies, Institute of Psychiatry, Psychology and Neuroscience, King’s Health Partners, London, UK; 2 Cambridgeshire and Peterborough NHS Foundation Trust, Cambridge, UK; 3 Oxford Autoimmune Neurology Group, Nuffield Department of Clinical Neurosciences, University of Oxford, Oxford, UK; 4 Department of Psychiatry, University of Oxford, Oxford, UK; 5 Division of Clinical Neurology, John Radcliffe Hospital, Oxford, UK; 6 Department of Clinical Neurophysiology, John Radcliffe Hospital, Oxford, UK; 7 Epilepsy Research Group, Nuffield Department of Clinical Neurosciences, Oxford University, John Radcliffe Hospital, Oxford, UK; 8 Department of Neuroradiology, King’s College Hospital NHS Foundation Trust, London, UK; 9 Department of Radiology Guy’s, St Thomas’ NHS Foundation Trust, London, UK; 10 Department of Biostatistics and Health Informatics, Institute of Psychiatry, Psychology and Neuroscience, King’s College London, London, UK; 11 UCL Institute of Mental Health, University College London, London, UK

**Keywords:** Antineuronal antibodies, Autoimmune psychosis, Autoimmune encephalitis, Meta-analysis, NMDAR, Psychosis

## Abstract

**Background:**

A variety of psychiatric syndromes are associated with NMDAR autoantibodies; however, their clinical relevance when only present in the serum is unclear. We explored whether patients with CSF NMDAR autoantibodies could be distinguished from patients with serum-only NMDAR autoantibodies.

**Methods:**

The electronic databases MEDLINE, EMBASE, PubMed, and PsycINFO were searched. Articles reporting adult patients with isolated psychiatric features and positive for NMDAR autoantibodies with relevant investigations were included. Patient level meta-analysis compared patients positive for CSF NMDAR autoantibodies with patients positive for serum NMDAR autoantibodies, but negative for CSF NMDAR autoantibodies. Dichotomous data were analysed using crude odds ratios (OR), whilst continuous data were analysed using Mann–Whitney Test (U). The protocol was prospectively registered (CRD42018082210).

**Results:**

Of 4413 publications, 42 were included, reporting 79 patients. Median age was 34 years (IQR 19 years); 56% (45/79) were female and 24% (16/68) had a tumour. In total, 41 patients were positive for CSF autoantibodies and 20 were positive for serum-only autoantibodies. Patients with CSF autoantibodies were significantly more likely to be female (*p* < 0.001) and have a rapid (< 3 month) onset of symptoms (*p* = 0.02) than patients with serum-only autoantibodies. They were also more likely to present with psychosis (*p* < 0.001), exhibit EEG (*p* = 0.006), MRI (*p* = 0.002), and CSF (*p* = 0.001) abnormalities, but less likely to present with insomnia (*p* = 0.04).

**Conclusions:**

Patients with an isolated psychiatric syndrome with CSF NMDAR autoantibodies can potentially be distinguished from those with serum-only NMDAR autoantibodies based on clinicodemographic and investigation findings.

**Supplementary Information:**

The online version contains supplementary material available at 10.1007/s00415-022-11224-6.

## Introduction

NMDAR-antibody encephalitis (NMDARE) is a serious yet potentially reversible autoimmune neuropsychiatric disorder associated with IgG autoantibodies against the NR1 subunit of the N-methyl-D-aspartate receptor (NMDAR) [8]. It predominantly affects young female adults, and around 20% of cases are associated with ovarian teratomas [38]. The clinical syndrome is multi-stage, with an initial prodromal phase followed by prominent psychiatric symptoms, cognitive impairment, movement disorders, seizures, and in severe cases reduced consciousness and hypoventilation [7]. According to international consensus criteria [11], a diagnosis of definite NMDARE requires the rapid onset (< 3 months) of one, or more major groups of symptoms coinciding with detection of IgG GluN1 autoantibodies in the cerebrospinal fluid (CSF). Observational evidence indicates that early treatment with immunotherapy is associated with significantly improved clinical and functional outcomes [38].

Among patients fulfilling the diagnosis of NMDARE, 4–5% have an isolated psychiatric syndrome [1, 18] usually encompassing features of psychosis, mood disorders, and catatonia [1]. This has prompted psychiatrists to explore whether a proportion of patients with a primary psychiatric diagnosis may have an NMDAR-antibody mediated disorder. Observational studies of patients with a primary psychiatric diagnosis have identified NMDAR autoantibodies in the serum of a subgroup of patients [2, 43]; however, these patients have been found to be clinically indistinguishable from those who are antibody-negative [21], and antibody prevalence rates may be similar to the general population [6, 10].

Definite diagnosis of NMDARE relies heavily on the detection of CSF NMDAR autoantibodies. As awareness of the disorder being closely associated with psychiatric symptoms has increased, CSF and serum testing among patients with a purely psychiatric syndrome is increasingly commonplace. Within a psychiatric context, antibody testing is overwhelmingly performed on serum samples [5]. However, the clinical relevance of serum NMDAR autoantibodies without accompanying CSF NMDAR autoantibodies is unclear.

Using a systematic review and meta-analysis approach, we explored whether patients with an isolated psychiatric syndrome with serum-only NMDAR autoantibodies differed from patients with CSF NMDAR autoantibodies based on their clinical and investigation findings.

## Methods

### Search strategy and selection criteria

We adhered to the Preferred Reporting Items for Systematic Reviews and Meta-Analyses (PRISMA) guidelines and registered the protocol on PROSPERO (CRD42018082210). The electronic databases MEDLINE, EMBASE, PubMed, and PsycINFO were searched for observational studies published in English between January 2006 and May 2020 using the search terms: (antibod* OR auto-antibod* OR autoantibod*) AND (N-methyl-D-aspartate or NMDA*). The database search was supplemented by reviewing the references of included publications and consulting with experts in the field. We restricted studies to those which reported individual patient-level data.

Inclusion criteria were: (i) a diagnosis of a psychiatric disorder, or presence of at least one psychiatric symptom as listed in the Brief Psychiatric Rating Scale [26]. This instrument was chosen as it covers the breadth of psychopathology associated with NMDARE [1, 38], (ii) NMDAR-antibody positivity in the CSF and/or serum using any assay type, and (iii) investigation with structural MRI and an awake EEG, coinciding with antibody positivity. Whilst we were also interested in CSF findings (other than NMDAR-antibody status), we did not stipulate this as an inclusion criterion due to low anticipated rates among patients.

Exclusion criteria were (i) the presence of new neurological or dysautonomic features, unless they could be accounted for by the psychiatric syndrome or medication. This criterion was applied to identify patients most likely to present to psychiatric services. In addition, (ii) patients under 18 years at the time of antibody detection were excluded based on the lower reported prevalence of psychiatric features [38] and difficulties in reliably assessing psychopathology in this group. Patients with additional autoantibodies were included, as were patients with a pre-existing neurological disorder.

Two authors (GB and MFL) screened the titles and abstracts of identified articles. Full articles of the remaining studies were reviewed to confirm eligibility. If more than one publication reported the same case, the more comprehensive was selected. Differences were resolved by a third author (TP).

### Data collection and synthesis

A piloted standardised data-extraction form was used (see supplemental section Table 3 for a full list of variables collected). Where data were missing, authors were contacted via email. The following variables were extracted (a) demographics, including sex and age; (b) antibody findings; (c) psychopathology; (d) symptom duration; (e) CSF, EEG, and MRI abnormalities; and (f) treatment and response. Rapid onset of symptoms was defined a priori as a duration of less than 3 months, at time of antibody detection, in line with established consensus criteria [11].

Psychopathology was appraised against the following broad symptom domains associated with NMDARE [1, 38]: psychosis, mood, catatonia, sleep, and cognitive impairment. Where a patient was assigned a psychiatric diagnosis, without detailing the underlying psychiatric symptoms, psychopathology was based upon the core features of that disorder, as defined by DSM-5 [3]. Investigation findings were classified as either normal or abnormal, with abnormalities further categorised. Risk of bias for each study was assessed using an 11-item tool adapted from the National Institute of Clinical Excellence [35], and PRISMA guidelines [15]. Each study was categorised as being at either low (> 8 points), medium (4–7 points), or high (< 4 points) risk of bias based on the total score.

### Statistical analysis

Following initial summary statistics, patients with CSF NMDAR autoantibodies were compared to patients with serum-only NMDAR autoantibodies using a patient-level meta-analytic approach. Analysis was restricted to patients who had undergone CSF antibody testing, to confirm the presence or absence of detectable CSF autoantibodies. The primary comparisons were demographic and clinical characteristics and investigation abnormalities. Secondary comparisons were treatment and clinical outcome, the later categorised as full, partial or no improvement. Due to the uncertainty regarding the pathogenic potential of non-IgG autoantibodies, a sensitivity analysis was performed with the analysis repeated and restricted to patients with confirmed IgG autoantibodies.

Crude odds ratios (OR) with 95% confidence intervals (CI) were estimated for dichotomous variables. A Mann–Whitney *U* Test was performed for continuous variables. Significance level was *p* ≤ 0·05 for all analyses. Given the exploratory nature, multiple comparison correction was not performed. Missing observations were handled through pairwise deletion. Planned analyses were developed in collaboration with a senior statistician (BC). Statistical analysis and visualisation was performed using R (Version 4.0.3; R Core Team 2020).

## Results

### Literature search

A total of 4413 publications were identified through electronic databases using prespecified search terms and a further 13 via other sources. After screening the titles and abstracts, 517 publications were fully reviewed. A total of 42 studies satisfied inclusion criteria, reporting a total of 79 patients (see online supplemental Fig. 1 for PRISMA flowchart and see online supplemental Table 1 for references of included papers). Publication year ranged from 2011 to 2020 and publication type were research papers (*N* = 35) and abstracts (*N* = 7). Studies were conducted across 17 countries; the most common were USA (*N* = 13), Japan (*N* = 4), UK (*N* = 3), Spain (*N* = 2), China (*N* = 2), and France (*N* = 2). The most frequent study designs were case reports (*N* = 25), case series (*N* = 7) and case–control (*N* = 6) studies, which accounted for 90% of studies (see supplemental Table 1 for study characteristics).

**Fig. 1:**
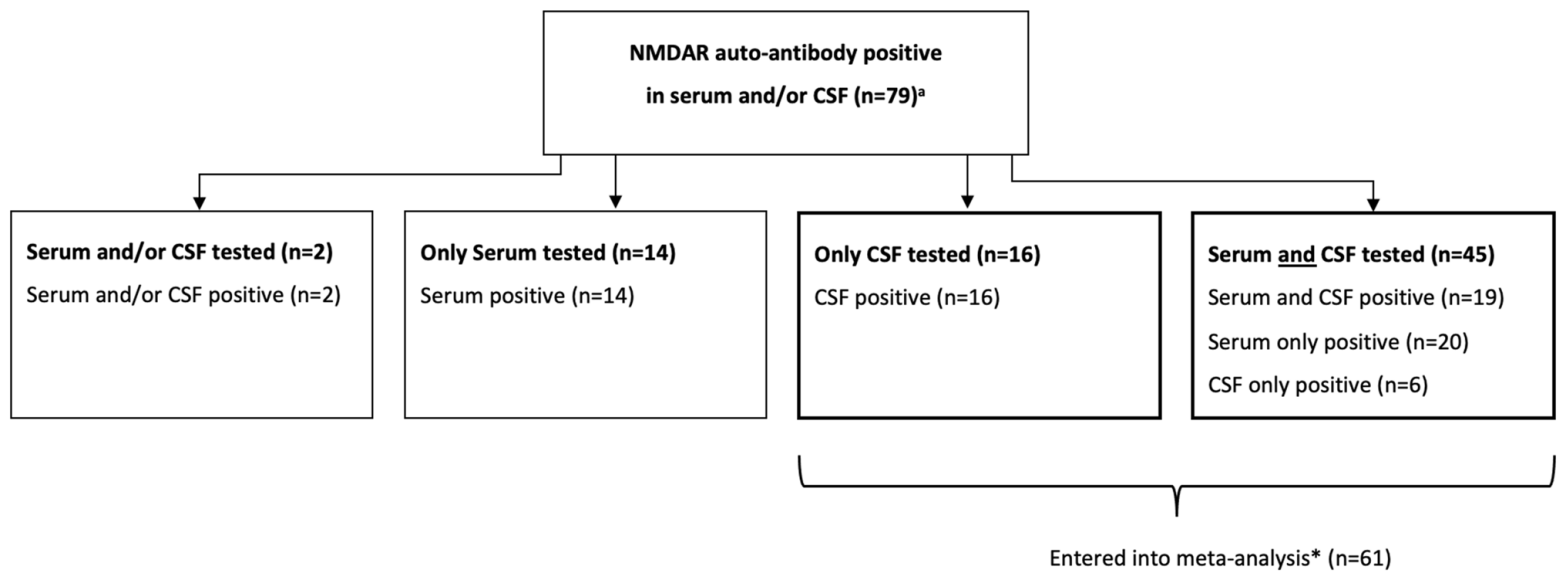
Overview of antibody detection. Only cases where CSF testing was performed and where NMDAR auto-antibody assays were positive in either CSF and/or serum were included in the meta-analysis. In 2 cases, it was unclear whether autoantibodies were detected in the serum and/or CSF

**Table 1:** Summary demographics, clinical features, investigation findings, treatment, and outcome by NMDAR-antibody status

	CSF or serum NMDAR-antibody positive (*N* = 79)	CSF NMDAR-antibody status confirmed (*n* = 61)		
		CSF positive (*n* = 41)	Serum-only positive (*n* = 20)	*P* value
Age (median years)^†^	34 (19)	32 (21)	30 (15)	0.55
Symptom duration (weeks)^†^	12 (229)	4 (11)	260 (884)	0.03*
Diagnosis				
Psychiatric diagnosis	48/63 (76%)	15/28 (54%)	19/19 (100%)	0.11
Neurological diagnosis	15/63(24%)	13/28 (46%)	0/19 (0%)	0.11
Definite NMDARE	41/79 (52%)	41/41 (100%)	0/20 (0%)	0.05*
EEG				
Any abnormality	33/61 (54%)	25/41 (61%)	8/20 (40%)	< 0.01**
Generalised slowing	16/68 (24%)	9/30 (30%)	2/20 (10%)	0.06
Focal slowing	12/68 (18%)	7/30 (23%)	3/20 (15%)	0.35
Epileptiform activity	3/68 (4%)	1/30 (3%)	2/20 (10%)	0.46
CNS medication	34/58 (58%)	17/24 (71%)	11/17(65%)	0.54
MRI				
Any abnormality	21/61 (34%)	19/41 (54%)	2/20 (10%)	< 0.01**
Hyperintensity	12/74 (16%)	8/36 (22%)	1/20 (5%)	0.16
Temporal lobe hyperintensity	5/74 (7%)	4/36 (11%)	0/20 (0%)	0.45
Atrophy	7/74 (9%)	2/36 (6)	0/20 (0%)	0.66
CSF				
Any abnormality	32/46 (67%)	25/31 (81%)	7/15 (47%)	< 0.01**
Elevated protein	15/29 (52%)	8/18 (44%)	6/8 (75%)	0.14
Oligoclonal bands	8/27 (30%)	4/14 (29%)	2/10 (20%)	0.63
Pleocytosis	13/35 (37%)	10/18(56%)	1/15 (7%)	0.03*
Clinical improvement	63/66 (95%)	37/40 (93%)	18/18 (100%)	0.60
Follow-up (median years)	1.1(1.5)	1.3 (1.7)	0.9 (0.4)	0.48
Relapse	2/53(4%)	2/36 (6%)	0/11 (0%)	0.84
Received immunotherapy	44/65 (68%)	36/40 (90%)	4/17 (24%)	< 0.001***
Clinical improvement with immunotherapy	37/41 (90%)	31/33 (94%)	3/4 (75%)	0.38

Unless specified, data are categorical with the proportion of patients identified as having the feature present (%) with p values calculated by likelihood ratio. Continuous data (†) with median and interquartile range with *p* values calculated by Mann–Whitney *U* test. ^a^Insufficient data to calculate interquartile range. Improvement with immunotherapy defined as partial or full remission of symptoms. Comparison of relapse not performed due to absence of positive cases. Significance is denoted as follows: *p < 0.05, **p < 0.01,***p < 0.001. For full set of data, see Supplementary Table 3.]

### Risk-of-bias assessment

Most studies were retrospective single-centre case studies, or series. The majority did not prespecify outcomes, report consecutive cases, or stratify outcomes by confounders. Consequently, most studies were rated as being at high risk of bias (*n* = 32), with a minority at medium (*n* = 9) or low risk of bias (*n* = 2) (see supplemental section Table 2 for further details).

### Antibody results

In total, 79 patients were identified with a median age of 34 years (interquartile range 19 years), with 45 (57%) being female and 16/68 (24%) having a tumour reported. Of these, 61 (77%) patients underwent CSF NMDAR-antibody testing with 41(67%) found to be CSF antibody positive. Cell-based assay (either live or fixed) was performed in all cases where assay type was reported (n = 48) and IgG autoantibodies were detected in all cases where antibody class was reported (*n* = 25). Of the 61 patients who underwent serum NMDAR-antibody testing, 55/61 (90%) were seropositive. Cell-based assay was performed in all cases where assay type was reported (*n* = 50). Where serum antibody class was reported, 40/42 (95%) patients had IgG autoantibodies detected. For CSF and serum NMDAR-antibody testing, very few cases reported the type of cell-based assay performed (i.e., fixed or live cell-based).

A total of 32/79 (40%) patients underwent serum or CSF antibody testing and 45 (57%) patients underwent both (see Fig. 1 for overview of antibody detection). In two (3%) cases, it was unclear whether autoantibodies were present in the serum and/or CSF. Of patients who underwent both serum and CSF testing, 19/45 (42%) were antibody positive in CSF and serum, 6/45 (13%) were positive in CSF only, and 20/45 (44%) were positive in serum only. Subsequent analyses were restricted to the 61 patients who had undergone CSF antibody testing.

### Provisional diagnosis and clinical characteristics

**Fig. 2:**
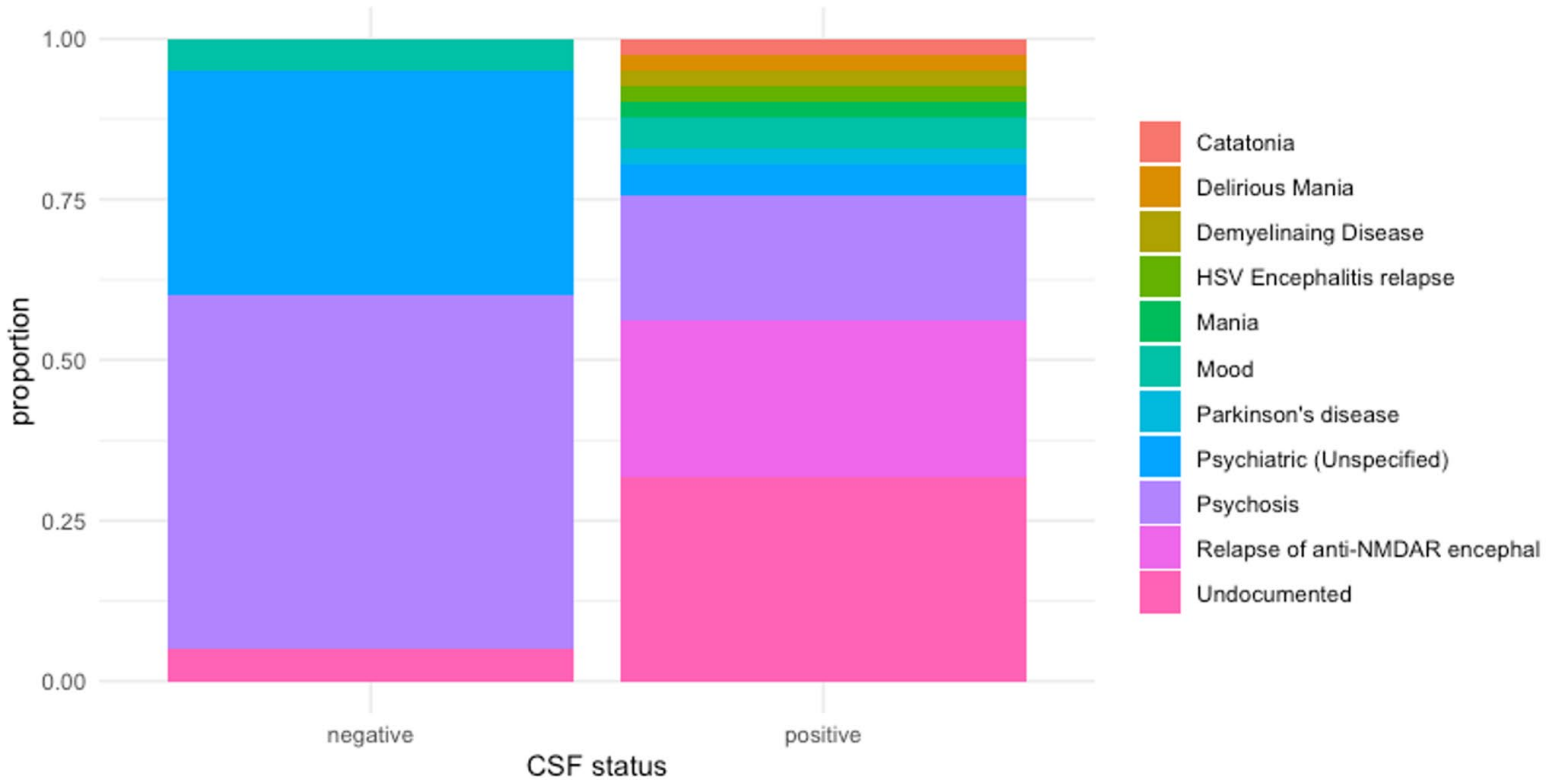
Provisional diagnosis of patients with CSF- and serum-only NMDAR autoantibodies

Figure 2 shows the provisional diagnosis of patients with CSF and serum-only NMDAR autoantibodies. Amongst patients with CSF NMDAR autoantibodies, the commonest diagnosis was a relapse of NMDARE (10/41; 24%), followed by psychosis (8/41; 20%). In patients with serum-only NMDAR autoantibodies, the commonest diagnosis was psychosis (11/20; 55%).

**Fig. 3:**
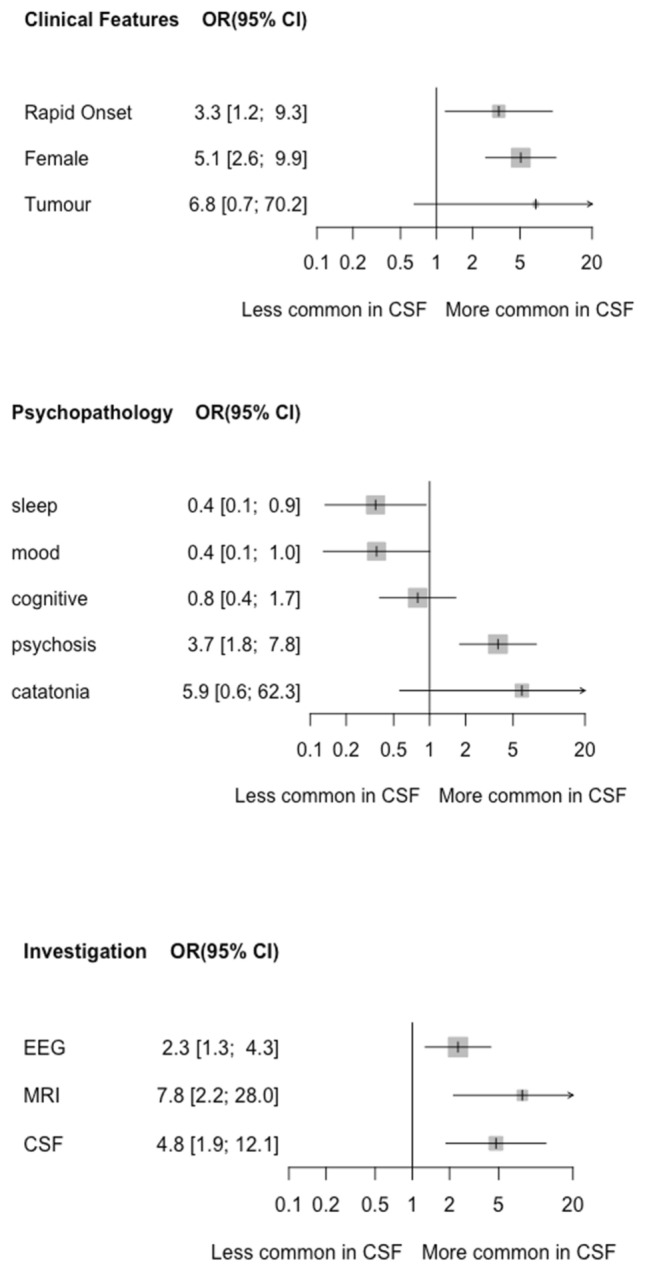
Forest plot of random-effects odds ratios (OR) and 95% confidence intervals comparing patients with CSF NMDAR autoantibodies to those with serum-only NMDAR autoantibodies according to **a** clinical and demographic characteristics, **b** psychiatric symptoms, and **c** investigation findings. CSF abnormalities were restricted to elevated protein, oligoclonal bands, and pleocytosis

Figure 3 and Table 1 compare the characteristics of patients with CSF and serum-only NMDAR autoantibodies. CSF autoantibodies were significantly associated with being female (OR 5.1, 95% CI 2.6–9.9, *p* < 0.001) and a rapid onset of symptoms (OR 3.3, 95% CI 1.2–9.3, *p* = 0.02). Tumours identified in CSF-positive patients were predominantly ovarian teratomas (*n* = 10). Adenocarcinoma of the colon (*n* = 1), phaeochromocytoma (*n* = 1), and neuroendocrine carcinoma (small cell tumour) and hepatocellular carcinoma (*n* = 1) were also reported. For serum-only NMDAR patients, only one tumour (ovarian Sertoli–Leydig tumour) was identified. Patients with CSF autoantibodies had a median symptom duration of 4 weeks and 48% (19/40) had a rapid onset of symptoms (< 3 months), thereby meeting criteria for definite NMDARE [11]. Patients with CSF NMDAR autoantibodies had a significantly shorter duration of symptoms than patients with serum NMDAR autoantibodies (*p* = 0.03). Patients with CSF NMDAR autoantibodies were significantly more likely to have psychotic symptoms (OR 3.7, 95% CI 1.8–7.8, *p* < 0.001) and were significantly less likely to have sleep symptoms (OR 0.4, 95% CI 0.1–0.9, *p* = 0.04). There were no significant differences in the other symptom domains.

### Investigations

Patients with CSF NMDAR autoantibodies were more likely to have EEG abnormalities (OR = 2.3, 95% CI 1.3–4.3, *p* = 0.006) compared to patients with serum-only NMDAR autoantibodies. They were also more likely to have MRI abnormalities (OR 7.8, 95% CI 2.2–28.0, *p* = 0.002). The commonest MRI abnormality in patients with CSF autoantibodies was signal hyperintensity. Notably, in the only patient with serum-only NMDAR autoantibodies with hyperintensities, these were reported as non-specific T2 white matter lesions, rather than a focal area of cortical hyperintensity. Finally, we compared the frequency of CSF abnormalities (specifically, elevated protein, oligoclonal bands, and pleocytosis). Patients with CSF NMDAR autoantibodies were more likely to have one or more CSF abnormalities (OR = 4.8, 95% CI 1.9–12.1, *p* = 0.001), and were specifically more likely to have pleocytosis (> 5 leucocytes/μl) (*p* = 0.03).

### Treatment and outcome

Patients with CSF autoantibodies were more likely to receive immunotherapy compared to patients with serum-only NMDAR autoantibodies (*p* < 0.001). The commonest type of immunotherapy in patients with CSF autoantibodies was steroids (*n* = 31), followed by intravenous immunoglobulin (IVIG) therapy (*n* = 23). Median follow-up after immunotherapy in patients with CSF and serum-only NMDAR autoantibodies was 1.3 and 0.9 years, respectively, and did not differ significantly between groups (*p* = 0.48). There was no difference in response to immunotherapy, or likelihood of relapse between groups; however, only a small number of patients with serum-only NMDAR autoantibodies received immunotherapy (*n* = 4).

As part of a planned sensitivity analysis, we repeated the analysis restricted to patients with confirmed IgG autoantibodies (*n* = 25). Significant associations with CSF NMDAR autoantibodies were unchanged, with the exception of rapid onset (*p* = 0.23), tumour (*p* = 0.14), and symptom duration (*p* = 0.09), which became non-significant.

## Discussion

Through a systematic review and patient-level meta-analysis, we identified 79 patients with an isolated psychiatric syndrome associated with NMDAR autoantibodies who had undergone neurological investigation. Exploratory analysis revealed patients with CSF autoantibodies differed from those with serum-only autoantibodies on several demographic, clinical, and investigation finding variables.

With respect to demographic and clinical profile, patients with CSF NMDAR autoantibodies were more likely to be female and to have a rapid onset of symptoms. These clinical characteristics are consistent with the epidemiology of NMDARE, a disorder based on CSF NMDAR-antibody detection [7]. In the largest observational study of NMDARE to date, 81% were female, and 38% had a tumour reported (most commonly an ovarian teratoma) [38], which was a similar proportion to the CSF antibody-positive group in this study. According to current consensus criteria, all of the patients we identified with CSF NMDAR autoantibodies met the criteria for definite NMDARE and 94% of those treated with immunotherapy showed an improvement.

Patients with CSF autoantibodies were similar to patients with serum-only NMDAR autoantibodies across several psychiatric domains; however, there were some differences. Notably, patients with CSF NMDAR autoantibodies were more likely to exhibit psychotic symptoms. The finding is consistent with a recent systematic review of patients with NMDARE, which identified a consistent and distinctive psychopathology including psychotic symptoms [1].

In terms of investigation findings, patients with CSF autoantibodies were more likely to have EEG abnormalities. These were most commonly focal or generalised slowing, which are consistent with NMDARE [38]. Patients with CSF autoantibodies were also more likely to have additional CSF abnormalities, particularly elevated white cells (pleocytosis). Almost half of patients with serum-only NMDAR autoantibodies had one or more CSF abnormalities, most commonly elevated protein. It has been hypothesised that the brain may act as an immunoprecipitator, leading autoantibodies to be undetectable in the CSF [9]. In these situations, disruption of the blood–brain barrier would be necessary to permit autoantibodies synthesised in the serum to transfer into the CSF and allow binding to NMDA receptors in the brain [31]. Further research measuring blood and CSF autoantibodies, alongside indices of blood–brain barrier integrity, are warranted. In a naturalistic study of 124 patients with an isolated psychotic syndrome who underwent CSF analysis, 18 (14.6%) had intrathecal oligoclonal bands and 6 (4.8%) had pleocytosis [27]. Our meta-analysis found that a higher proportion of patients with NMDAR autoantibodies had these abnormalities, including patients with serum-only NMDAR autoantibodies. This suggests that CNS immune activation may take place, in at least a subset of patients with detectable neuronal autoantibodies in serum only; however, it is beyond the scope of the study to infer causality. Also of clinical relevance was the finding that patients with CSF autoantibodies were more likely to have EEG and MRI abnormalities than patients with serum-only NMDAR autoantibodies, with an unadjusted odds ratio of 2.3 and 7.8, respectively.

Most patients with CSF NMDAR autoantibodies who received immunotherapy improved and did not relapse during follow-up; however, outcome measures and follow-up varied. This finding is consistent with evidence that NMDARE is responsive to prompt immunotherapy [38]. Intriguingly, three of the four patients with serum-only NMDAR autoantibodies treated with immunotherapy also improved and did not relapse (follow-up ranged between 6 months and 2 years), in keeping with a small open-label trial [43]. However, the small number of patients, absence of blinding, and possible publication bias limit further interpretation; randomised controlled trials are awaited with interest [22]. Interestingly, all the patients treated with antipsychotics, in the absence of immunotherapy, also improved [4, 16, 19, 24, 39–42]. This may reflect natural changes in symptom severity over time, or a response to antipsychotic medication. Antipsychotics are recognised to be immunomodulatory [34, 36], including having effects on the adaptive immune system [28]. Further research is indicated to explore whether these effects play a role in the treatment of antibody-associated psychiatric syndromes.

Previous studies of isolated psychiatric symptoms in patients with NMDAR autoantibodies have been largely limited to case reports or case series, and this is the first attempt to pool the existing literature using meta-analytic methods. There is robust evidence for the pathogenic potential of NMDAR autoantibodies when exposed to brain parenchyma at sufficient concentrations [16, 30]. Our study supports the consensus criteria that patients with new-onset psychiatric symptoms who are CSF positive for NMDAR autoantibodies can be considered to have a monosymptomatic form of NMDARE. Similarities in the clinical characteristics, investigation findings and response to treatment, with more typical presentations of NMDARE support this assertion. Whilst we excluded patients who exhibited neurological or autonomic features, we note that some patients may have gone on to develop these in the absence of immunotherapy [14]. This may be particularly relevant in patients who had a previously been diagnosed with NMDARE, who may have been initiated on treatment more promptly given their medical history.

Unlike a CSF NMDAR-antibody positive result, the clinical relevance of serum-only NMDAR-antibody positivity is less clear. The demographic and clinical characteristics were less comparable with NMDARE, and patients were less likely to exhibit the EEG, CSF, and MRI changes associated with NMDARE, or encephalitis more generally. This is consistent with a recent large prospective study, in which patients with psychosis who were seropositive for NMDAR autoantibodies did not differ in response to antipsychotic medication compared to patients who were seronegative [33]. However, we did identify higher rates of CSF, EEG, and MRI abnormalities than would normally be expected in patients with a primary psychiatric diagnosis [25, 29, 37], suggesting that, at least in a subgroup, serum autoantibodies may be an epiphenomena of a biologically relevant underlying process. However, a potential confounding factor is that patients who test positive for serum neuronal autoantibodies are more likely to under further investigations. Similarly, patients presenting with atypical symptoms or imaging findings may prompt clinicians to test for autoantibodies.

Whilst some studies have suggested increased seroprevalence of NMDAR autoantibodies in patients with psychiatric disorders [21, 32], this has not been consistently found [6, 10]. In a recent meta-analysis, the overall prevalence of NMDAR IgG antibody seropositivity was estimated to be less than 1% in patients with psychosis, and did not differ significantly compared to healthy individuals [5]. However, when restricted to studies using a live cell-based assay, the estimated prevalence increased to almost 3%, and patients were over four times more likely to be seropositive than healthy individuals, suggesting that assay type is an important mediator of antibody detection. Through sensitivity analyses, we were able to demonstrate that all our findings were robust when restricting to cases where NMDAR autoantibodies of IgG class was confirmed, except for the association between a postive CSF NMDAR-antibody result and rapid symptom onset, tumour presence and shorter symptom duration which became non-significant. However, due to very few cases reporting details of the assay performed, we were unable to explore the influence of assay type.

From a clinical perspective, the meta-analysis highlights the value of neurological investigation in patients presenting with psychiatric symptoms and NMDAR-antibody seropositivity. This is particularly relevant, given that routine screening for serum NMDAR autoantibodies is increasingly recommended in first-episode psychosis [12]. Lumbar puncture can confirm (or exclude) NMDARE, which is particularly important due to the association between prompt use of immunotherapy and outcome [38]. Furthermore, findings in this highly enriched sample of cases suggest that EEG and MRI are informative and carry the advantages of being non-invasive and well tolerated. Should these investigations detect a potentially relevant abnormality, the index of suspicion for autoimmune encephalitis should be raised. Given the current lack of availability of lumbar puncture facilities in most mental healthcare settings, further research using prospective methods is urgently needed to investigate the feasibility of developing a clinical tool that captures the complex psychopathology associated with NMDAR-antibody mediated disorders. In addition, alternative biomarkers that do not require CSF sampling, such as neurofilament light chain, may have clinical utility in identifying patients with NMDARE[13]. Such approaches would help clinicians identify which patients are in greatest need of CSF antibody testing [1].

Several limitations of the current study should be acknowledged. First, case reports and case series, which constituted the bulk of included studies, are generally considered low levels of evidence and may report novel or atypical presentations. Second, there is likely to be a degree of reporting bias, leading to patients with absent CSF NMDAR antibodies to be underreported in the literature as they do not meet criteria for NMDARE [11]. Third, there was a large degree of heterogeneity in detail; such as the granularity of psychopathology. We were partially able to address this by contacting authors. Nevertheless, there is likely to have been underreporting of clinical and investigation findings, highlighting the need for further research adopting a prospective design, so that patients are phenotyped in a standardised way. This is particularly relevant with regards the assay used, which vary in sensitivity to detect NMDAR autoantibodies [5]. Fourth, due to the reliance upon case report and case series, it is not possible to generate estimates of overall prevalence, or incidence. Future research in a larger sample with an appropriate patient control group (i.e., NMDAR-antibody negative) is recommended. Fifth, due to the interval from symptom onset to antibody testing among some patients, it is plausible that a proportion of cases categorised as serum-only NMDAR-antibody positive may have been CSF positive at an earlier stage. Sixth, few studies reported the titre of autoantibodies detected, which has been shown to relate to pathogenic potential [16, 17]. It is important to acknowledge that NMDARE with purely isolated psychiatric syndrome with CSF NMDAR autoantibodies is rare. Of the cases we identified, almost a quarter were a relapse. Finally, it is not possible to know how many of the included patients would have developed neurological or autonomic features associated with NMDAR-antibody encephalitis without immunotherapy. This may be particularly relevant to patients with CSF autoantibodies, who were more likely to undergo antibody testing sooner.

In conclusion, there is increasing recognition of psychiatric syndromes associated with neuronal autoantibodies, with important treatment implications. We found that patients with CSF NMDAR autoantibodies were clinically distinct from those with serum-only NMDAR autoantibodies based on their demographic and clinical characteristics. EEG and MRI were able to assist the differentiation. Patients with new-onset psychiatric syndromes are likely to be under the care of mental health services, and psychiatrists increasingly face the dilemma of whether to perform antibody screening. This meta-analysis supports existing evidence that a proportion of patients with new-onset psychiatric symptoms have NMDARE. Rapid onset of symptoms, female sex, and psychotic symptoms should raise the index of suspicion. CSF analysis is the definitive investigation to confirm the diagnosis; however, where lumbar puncture is not immediately feasible, our study suggests that EEG and MRI are informative.

## Supplementary Information

Below is the link to the electronic supplementary material.

Supplementary file1 (DOCX 119 KB)

## Data Availability

The datasets used and analysed during the current study are available in the Open Science Framework (OSF) Repository, https://osf.io/ajuw8/?view_only=4d5b5b31254e43f0813d7d3a368388fe.

## References

[1] Al-Diwani A, Handel A, Townsend L, Pollak T, Leite MI, Harrison PJ, Lennox BR, Okai D, Manohar SG, Irani SR (2019). The psychopathology of NMDAR-antibody encephalitis in adults: a systematic review and phenotypic analysis of individual patient data. Lancet Psychiatry.

[2] Al-Diwani A, Pollak TA, Langford AE, Lennox BR (2017). Synaptic and neuronal autoantibody-associated psychiatric syndromes: controversies and hypotheses. Front Psychiatry.

[3] American Psychiatric Association (2013). Diagnostic and statistical manual of mental disorders.

[4] Arboleya S, Clemente A, Deng S, Bedmar M, Salvador I, Herbera P, Cunill V, Vives-Bauza C, Haro J, Canellas F, Julia M (2016) Anti-NMDAR antibodies in new-onset psychosis. Positive results in an HIV-infected patient. Brain, Behavior, and Immunity 56 (pp 56–60), 2016 Date of Publication: 01 Aug 2016 56:56–60 10.1016/j.bbi.2016.03.011 26996305

[5] Cullen AE, Palmer-Cooper EC, Hardwick M, Vaggers S, Crowley H, Pollak TA, Lennox BR (2021). Influence of methodological and patient factors on serum NMDAR IgG antibody detection in psychotic disorders: a meta-analysis of cross-sectional and case-control studies. Lancet Psychiatry.

[6] Dahm L, Ott C, Steiner J, Stepniak B, Teegen B, Saschenbrecker S, Hammer C, Borowski K, Begemann M, Lemke S, Rentzsch K, Probst C, Martens H, Wienands J, Spalletta G, Weissenborn K, Stocker W, Ehrenreich H (2014). Seroprevalence of autoantibodies against brain antigens in health and disease. Ann Neurol.

[7] Dalmau J, Gleichman AJ, Hughes EG, Rossi JE, Peng X, Lai M, Dessain SK, Rosenfeld MR, Balice-Gordon R, Lynch DR (2008). Anti-NMDA-receptor encephalitis: case series and analysis of the effects of antibodies. Lancet Neurol.

[8] Dalmau J, Tuzun E, Wu HY, Masjuan J, Rossi JE, Voloschin A, Baehring JM, Shimazaki H, Koide R, King D, Mason W, Sansing LH, Dichter MA, Rosenfeld MR, Lynch DR (2007). Paraneoplastic anti-*N*-methyl-d-aspartate receptor encephalitis associated with ovarian teratoma. Ann Neurol.

[9] Ehrenreich H (2017). Autoantibodies against the *N*-Methyl-d-aspartate receptor subunit NR1: untangling apparent inconsistencies for clinical practice. Front Immunol.

[10] Gaughran F, Lally J, Beck K, McCormack R, Gardner-Sood P, Coutinho E, Jacobson L, Lang B, Sainz-Fuertes R, Papanastasiou E, Di Forti M, Nicholson T, Vincent A, Murray RM (2018). Brain-relevant antibodies in first-episode psychosis: a matched case-control study. Psychol Med.

[11] Graus F, Titulaer MJ, Balu R, Benseler S, Bien CG, Cellucci T, Cortese I, Dale RC, Gelfand JM, Geschwind M, Glaser CA, Honnorat J, Hoftberger R, Iizuka T, Irani SR, Lancaster E, Leypoldt F, Pruss H, Rae-Grant A, Reindl M, Rosenfeld MR, Rostasy K, Saiz A, Venkatesan A, Vincent A, Wandinger KP, Waters P, Dalmau J (2016). A clinical approach to diagnosis of autoimmune encephalitis. Lancet Neurol.

[12] Guasp M, Gine-Serven E, Maudes E, Rosa-Justicia M, Martinez-Hernandez E, Boix-Quintana E, Bioque M, Casado V, Modena-Ouarzi Y, Guanyabens N, Muriana D, Sugranyes G, Pacchiarotti I, Davi-Loscos E, Torres-Rivas C, Rios J, Sabater L, Saiz A, Graus F, Castro-Fornieles J, Parellada E, Dalmau J (2021). Clinical, neuroimmunologic, and CSF investigations in first episode psychosis. Neurology.

[13] Guasp M, Martín-Aguilar L, Sabater L, Bioque M, Armangué T, Martínez-Hernández E, Landa J, Maudes E, Borràs R, Muñoz-Lopetegi A, Saiz A, Castro-Fornieles J, Graus F, Parellada E, Querol L, Dalmau J (2022). Neurofilament light chain levels in anti-NMDAR encephalitis and primary psychiatric psychosis. Neurology.

[14] Gurrera RJ (2019). Frequency and temporal sequence of clinical features in adults with anti-NMDA receptor encephalitis presenting with psychiatric symptoms. Psychol Med.

[15] Higgins JP, Altman DG, Gotzsche PC, Juni P, Moher D, Oxman AD, Savovic J, Schulz KF, Weeks L, Sterne JA, Cochrane Bias Methods G, Cochrane Statistical Methods G (2011). The Cochrane Collaboration’s tool for assessing risk of bias in randomised trials. BMJ.

[16] Jezequel J, Johansson EM, Dupuis JP, Rogemond V, Grea H, Kellermayer B, Hamdani N, Le Guen E, Rabu C, Lepleux M, Spatola M, Mathias E, Bouchet D, Ramsey AJ, Yolken RH, Tamouza R, Dalmau J, Honnorat J, Leboyer M, Groc L (2017). Dynamic disorganization of synaptic NMDA receptors triggered by autoantibodies from psychotic patients. Nat Commun.

[17] Jezequel J, Johansson EM, Leboyer M, Groc L (2018). Pathogenicity of antibodies against NMDA receptor: molecular insights into autoimmune psychosis. Trends Neurosci.

[18] Kayser MS, Titulaer MJ, Gresa-Arribas N, Dalmau J (2013). Frequency and characteristics of isolated psychiatric episodes in anti-*N*-methyl-d-aspartate receptor encephalitis. JAMA Neurol.

[19] Kelleher E, McNamara P, Fitzmaurice B, Walsh R, Langan Y, Whitty P, Gill M, Vincent A, Doherty C, Corvin A (2015) Prevalence rate of *N*-methyl-d-aspartate (NMDA) receptor antibodies in first episode psychosis. European Psychiatry Conference: 23rd European Congress of Psychiatry, EPA 2015 Vienna Austria Conference Publication: (var pagings) 30 (pp 1568), 2015 Date of Publication: 31 Mar 2015 30:1568

[20] Kreye J, Wenke NK, Chayka M, Leubner J, Murugan R, Maier N, Jurek B, Ly LT, Brandl D, Rost BR, Stumpf A, Schulz P, Radbruch H, Hauser AE, Pache F, Meisel A, Harms L, Paul F, Dirnagl U, Garner C, Schmitz D, Wardemann H, Pruss H (2016). Human cerebrospinal fluid monoclonal *N*-methyl-d-aspartate receptor autoantibodies are sufficient for encephalitis pathogenesis. Brain.

[21] Lennox BR, Palmer-Cooper EC, Pollak T, Hainsworth J, Marks J, Jacobson L, Lang B, Fox H, Ferry B, Scoriels L, Crowley H, Jones PB, Harrison PJ, Vincent A (2017). Prevalence and clinical characteristics of serum neuronal cell surface antibodies in first-episode psychosis: a case-control study. Lancet Psychiatry.

[22] Lennox BR, Tomei G, Vincent SA, Yeeles K, Pollard R, Palmer-Cooper E, Jones P, Zandi MS, Coles A (2019). Study of immunotherapy in antibody positive psychosis: feasibility and acceptability (SINAPPS1). J Neurol Neurosurg Psychiatry.

[23] Makuch M, Wilson R, Al-Diwani A, Varley J, Kienzler AK, Taylor J, Berretta A, Fowler D, Lennox B, Leite MI, Waters P, Irani SR (2018). N-methyl-D-aspartate receptor antibody production from germinal center reactions: therapeutic implications. Ann Neurol.

[24] Masopust J, Tvaroh A, Pavelek Z, Valis M (2018). Encephalitis with anti-NMDA receptor antibodies: paraneoplastic or non-paraneoplastic?. Neuro Endocrinol Lett.

[25] O'Sullivan SS, Mullins GM, Cassidy EM, McNamara B (2006). The role of the standard EEG in clinical psychiatry. Hum Psychopharmacol.

[26] Overall JE, Gorham DR (1962). The brief psychiatric rating scale. Psychol Reports.

[27] Oviedo-Salcedo Tatiana, de Witte Lot, Kümpfel Tania, Kahn René S., Falkai Peter, Eichhorn Peter, Luykx Jurjen, Hasan Alkomiet (2018). Absence of cerebrospinal fluid antineuronal antibodies in schizophrenia spectrum disorders. British Journal of Psychiatry.

[28] Papa I, Saliba D, Ponzoni M, Bustamante S, Canete PF, Gonzalez-Figueroa P, McNamara HA, Valvo S, Grimbaldeston M, Sweet RA, Vohra H, Cockburn IA, Meyer-Hermann M, Dustin ML, Doglioni C, Vinuesa CG (2017). T(FH)-derived dopamine accelerates productive synapses in germinal centres. Nature.

[29] Paquet C, Magnin E, Wallon D, Troussiere AC, Dumurgier J, Jager A, Bellivier F, Bouaziz-Amar E, Blanc F, Beaufils E, Miguet-Alfonsi C, Quillard M, Schraen S, Pasquier F, Hannequin D, Robert P, Hugon J, Mouton-Liger F, For e PLMn, collaborators (2016). Utility of CSF biomarkers in psychiatric disorders: a national multicentre prospective study. Alzheimers Res Ther.

[30] Planaguma J, Leypoldt F, Mannara F, Gutierrez-Cuesta J, Martin-Garcia E, Aguilar E, Titulaer MJ, Petit-Pedrol M, Jain A, Balice-Gordon R, Lakadamyali M, Graus F, Maldonado R, Dalmau J (2015). Human *N*-methyl d-aspartate receptor antibodies alter memory and behaviour in mice. Brain.

[31] Pollak TA, Drndarski S, Stone JM, David AS, McGuire P, Abbott NJ (2018). The blood-brain barrier in psychosis. Lancet Psychiatry.

[32] Pollak TA, McCormack R, Peakman M, Nicholson TR, David AS (2014). Prevalence of anti-*N*-methyl-d-aspartate (NMDA) receptor [corrected] antibodies in patients with schizophrenia and related psychoses: a systematic review and meta-analysis. Psychol Med.

[33] Pollak TA, Vincent A, Iyegbe C, Coutinho E, Jacobson L, Rujescu D, Stone J, Jezequel J, Rogemond V, Jamain S, Groc L, David A, Egerton A, Kahn RS, Honnorat J, Dazzan P, Leboyer M, McGuire P (2021). Relationship between serum NMDA receptor antibodies and response to antipsychotic treatment in first-episode psychosis. Biol Psychiat.

[34] Ponsford M, Castle D, Tahir T, Robinson R, Wade W, Steven R, Bramhall K, Moody M, Carne E, Ford C, Farewell D, Williams P, El-Shanawany T, Jolles S (2019). Clozapine is associated with secondary antibody deficiency. Br J Psychiatry.

[35] Reynolds TM, National Institute for H, Clinical E, Clinical Scince Reviews Committee of the Association for Clinical B (2006). National Institute for Health and Clinical Excellence guidelines on preoperative tests: the use of routine preoperative tests for elective surgery. Ann Clin Biochem.

[36] Robichon K, Patel V, Connor B, La Flamme AC (2020). Clozapine reduces infiltration into the CNS by targeting migration in experimental autoimmune encephalomyelitis. J Neuroinflammation.

[37] Sommer IE, de Kort GA, Meijering AL, Dazzan P, Hulshoff Pol HE, Kahn RS, van Haren NE (2013). How frequent are radiological abnormalities in patients with psychosis? A review of 1379 MRI scans. Schizophr Bull.

[38] Titulaer MJ, McCracken L, Gabilondo I, Armangue T, Glaser C, Iizuka T, Honig LS, Benseler SM, Kawachi I, Martinez-Hernandez E, Aguilar E, Gresa-Arribas N, Ryan-Florance N, Torrents A, Saiz A, Rosenfeld MR, Balice-Gordon R, Graus F, Dalmau J (2013). Treatment and prognostic factors for long-term outcome in patients with anti-NMDA receptor encephalitis: an observational cohort study. Lancet Neurol.

[39] Tsutsui K, Kanbayashi T, Takaki M, Omori Y, Imai Y, Nishino S, Tanaka K, Shimizu T (2017). *N*-Methyl-d-aspartate receptor antibody could be a cause of catatonic symptoms in psychiatric patients: case reports and methods for detection. Neuropsychiatr Dis Treat.

[40] Tsutsui K, Kanbayashi T, Tanaka K, Boku S, Ito W, Tokunaga J, Mori A, Hishikawa Y, Shimizu T, Nishino S (2012). Anti-NMDA-receptor antibody detected in encephalitis, schizophrenia, and narcolepsy with psychotic features. BMC Psychiatry.

[41] Warren N, Swayne A, Siskind D, O’Gorman C, Prain K, Gillis D, Blum S (2020). Serum and CSF Anti-NMDAR antibody testing in psychiatry. J Neuropsychiatry Clin Neurosci.

[42] Yoshimura B, Yada Y, Horigome T, Kishi Y (2015). Anti-*N*-Methyl-d-aspartate receptor encephalitis presenting with intermittent catatonia. Psychosomatics.

[43] Zandi MS, Irani SR, Lang B, Waters P, Jones PB, McKenna P, Coles AJ, Vincent A, Lennox BR (2011). Disease-relevant autoantibodies in first episode schizophrenia. J Neurol.

