# Supplementary material for: The clinical relevance of serum versus CSF NMDAR autoantibodies associated exclusively with psychiatric features: a systematic review and meta-analysis of individual patient data
Source: J Neurol. 2022 Jul 5;269(10):5302–11. doi: 10.1007/s00415-022-11224-6 (PMC9467941; doi:10.1007/s00415-022-11224-6)
Supplement: Supplementary file 1 — Supplementary file1 (DOCX 119 KB) [file 415_2022_11224_MOESM1_ESM.docx]

**The clinical relevance of serum versus CSF NMDAR autoantibodies associated exclusively with psychiatric features: a systematic review and meta-analysis of individual patient data**

**Supplemental Section**

Supplemental Figure 1: PRISMA flow chart

Supplemental Table 1: Summary of eligible publications

Supplemental Table 2. Assessment of the quality and risk of bias of included studies

Supplemental Table 3: Characteristics of included patients

Supplemental Section references

Supplemental Figure 1: PRISMA diagram depicting the flow of citations reviewed during the systematic review

Full-text articles excluded:

(n = 475)

Neurological symptoms

(n = 230)

No EEG (n = 60)

Unable to obtain full text (n = 2)

No individual patient characteristics (n = 39)

Paediatric cases (n = 4)

Article in Japanese (n = 1)

Additional records identified through other sources
(n = 13)

Studies included (n = 42)

Full-text articles assessed for eligibility
(n = 517)

Records excluded
(n = 3,004)

Records screened
(n = 3,521)

Records after duplicates removed
(n = 3,521)

Records identified through database searching
(n = 4,413)

Supplemental Table 1: summary of eligible publications

| Author | Year | Journal | Journal Specialty | Publication Type | Study design | Study location | Included Cases |
| --- | --- | --- | --- | --- | --- | --- | --- |
| Kung et al[ s19] | 2011 | Psychosomatics | Psychiatry | paper | case report | USA | 1 |
| Tsutsui et al^[s41]^ | 2012 | BioMed Central Psychiatry | Psychiatry | paper | case series | Japan | 1 |
| Hopkins et al^[s11]^ | 2013 | BMJ Case Reports | General | paper | case report | UK | 1 |
| Kayser et al^s^[16] | 2013 | JAMA Neurology | Neurology | paper | cohort | USA | 13 |
| Steiner et al^s^[39] | 2013 | JAMA Psychiatry | Psychiatry | paper | case-control | Germany | 12 |
| Leypoldt et al^s^[23] | 2013 | Neurology | Neurology | paper | case report | Spain | 1 |
| Kuppuswamy et al^s^[20] | 2014 | Gen Hosp Psychiatry | Psychiatry | paper | case series | USA | 1 |
| Attalla et al^s^[2] | 2014 | Journal of General Internal Medicine | Medicine | abstract | case report | USA | 1 |
| Kattepur et al^[s15]^ | 2014 | World Journal of Surgical Oncology | Surgery | paper | case report | India | 1 |
| Heresco Levy et al^[s10]^ | 2015 | Biological Psychiatry | Psychiatry | paper | case report | USA | 1 |
| Lu et al^[s25]^ | 2015 | BMJ Case Reports | General | paper | case report | USA | 1 |
| Kelleher et al^s^[17] | 2015 | European Psychiatry | Psychiatry | abstract | cohort | Ireland | 4 |
| Senda et al^s^[37] | 2015 | Journal of Clinical Psychopharmacology | Psychiatry | paper | case report | Japan | 1 |
| Grebenciucova et al^s^[8] | 2015 | Neurology | Neurology | abstract | case report | USA | 1 |
| Yoshimura et al^s^[42] | 2015 | Psychosomatics | Psychiatry | paper | case series | Japan | 2 |
| Kruse et al^s^[18] | 2015 | Psychosomatics | Psychiatry | paper | case-control | USA | 1 |
| Lalanne et al^s^[21] | 2015 | Journal of Neuropsychiatry and Clinical Neurosciences | Psychiatry | paper | case report | France | 1 |
| Mimbella et al^s^[28] | 2016 | Annual Assembly of the American Academy of Physical Medicine and Rehabilitation | Rehabilitation Medicine | abstract | case report | USA | 1 |
| Arboleya et al^s^[1] | 2016 | Brain, Behavior, and Immunity | Psychoneuroimmunology | paper | case-control | Spain | 1 |
| McKeon et al^s^[27] | 2016 | Journal of the International Neuropsychological Society | Neuropsychology | paper | case-control | Australia | 2 |
| Chen et al^s^[4] | 2016 | Medical Journal of Chinese People's Liberation Army | Medicine | paper | case series | China | 1 |
| Parfene et al^s^[30] | 2016 | Journal of Neuropsychiatry and Clinical Neurosciences | Psychiatry | paper | case report | USA | 1 |
| Rong et al^s^[35] | 2017 | BMC Psychiatry | Psychiatry | paper | case report | China | 1 |
| Foff et al^s^[7] | 2017 | Clincal EEG and Neuroscience | Clinical Neuroscience | paper | case-control | USA | 1 |
| Lim et al^s^[24] | 2017 | Journal of Clinical Neuroscience | Clinical Neuroscience | paper | case report | Singapore | 1 |
| Jezequel et al^s^[12] | 2017 | Nature Communications | Natural Sciences | paper | case-control | France | 5 |
| Tsutsui et al^s^[40] | 2017 | Neuropsychiatric Disease and Treatment | Psychiatry | paper | case series | Japan | 1 |
| Kar et al^s^[14] | 2017 | Progress in Psychiatry and Neurology | Psychiatry/  Neurology | paper | case report | UK | 1 |
| Dang-Vu et al^s^[5] | 2017 | Wisconsin Medical Journal | Medicine | abstract | case report | USA | 1 |
| Hanagasi et al^s^[9] | 2018 | Alzheimer’s & Dementia | Psychiatry | abstract | Case report | Turkey | 1 |
| Scott et al^s^[36] | 2018 | BJPsych Open | Psychiatry | paper | cohort | Australia | 1 |
| Masopust et al^s^[26] | 2018 | Neuro Endocrinology Letters | Neuroendocrinology | paper | case report | Czech Republic | 1 |
| Perero et al^s^[33] | 2018 | Neurology | Neurology | abstract | case series | USA | 1 |
| Blackman et al^s^[3] | 2018 | Psychological Medicine | Psychiatry | paper | case report | UK | 1 |
| Park et al^s^[31] | 2019 | Clinical Neurology and Neurosurgery | Clinical Neuroscience | paper | case report | South Korea | 1 |
| Laurikainen et al^s^[22] | 2020 | BMC Psychiatry | Psychiatry | paper | case report | Finland | 1 |
| Endres et al^s^[6] | 2020 | Frontiers in Neurology | Neurology | paper | case report | Germany | 1 |
| Warren et al^s^[29] | 2020 | Journal of Neuropsychiatry and Clinical Neurosciences | Psychiatry | paper | case series | Australia | 6 |
| Kaddu- Mulindwa et al^s^[13] | 2020 | Journal of NeuroVirology | Neurology | paper | case report | Germany | 1 |
| Sinani et al^s^[38] | 2020 | Multiple Sclerosis and Related Disorders | Neurology | paper | case report | Oman | 1 |
| Park et al^s^[32] | 2020 | Parkinsonism and Related Disorders | Neurology | paper | case report | South Korea | 1 |
| Restrepo-Martínez et al^s^[34] | 2020 | Psychosomatics | Psychiatry | paper | case report | Mexico | 1 |

Supplemental Table 2. Assessment of the quality and risk of bias of included studies

| Author | Year | Multi centre | Clear aims | Inclusion/  exclusion clear | Outcomes defined clearly | Recruitment period defined | Sample size  >50 | Prospective | Consecutive | Characteristics & treatment described | Investigation Findings described | Outcomes stratified by confounders | Score | Risk of Bias Rating |
| --- | --- | --- | --- | --- | --- | --- | --- | --- | --- | --- | --- | --- | --- | --- |
| Kung et al | 2011 | no | no | no | no | no | no | no | no | yes | yes | no | *2* | *high* |
| Tsutsui et al | 2012 | yes | no | no | no | yes | no | no | no | yes | yes | no | *4* | *medium* |
| Hopkins et al | 2013 | no | no | no | no | no | no | no | no | yes | yes | no | *2* | *high* |
| Leypoldt et al | 2013 | no | no | no | no | no | no | no | no | yes | yes | no | *2* | *high* |
| Kayser et al | 2013 | yes | yes | yes | yes | no | no | yes | yes | yes | yes | no | *8* | *low* |
| Steiner et al | 2013 | no | yes | yes | yes | yes | no | no | no | yes | yes | no | *6* | *medium* |
| Attalla et al | 2014 | no | no | no | no | no | no | no | no | yes | yes | no | *2* | *high* |
| Kattepur et al | 2014 | no | no | no | no | no | no | no | no | yes | yes | no | *2* | *high* |
| Kuppuswamy et al | 2014 | no | no | no | no | no | no | no | no | yes | yes | no | *2* | *high* |
| Grebenciucova et al | 2015 | no | no | no | no | no | no | no | no | yes | yes | no | *2* | *high* |
| Heresco Levy et al | 2015 | no | no | no | no | no | no | no | no | yes | yes | no | *2* | *high* |
| Kruse et al | 2015 | no | yes | yes | yes | yes | no | no | no | yes | yes | no | *6* | *medium* |
| Lalanne et al | 2015 | no | no | no | no | no | no | no | no | yes | yes | no | *2* | *high* |
| Lu et al | 2015 | no | no | no | no | no | no | no | no | yes | yes | no | *2* | *high* |
| Senda et al | 2015 | no | no | no | no | no | no | no | no | yes | yes | no | *2* | *high* |
| Yoshimura et al | 2015 | no | no | no | no | no | no | no | no | yes | yes | no | *2* | *high* |
| Kelleher et al | 2015 | no | yes | yes | no | no | no | yes | yes | no | yes | no | *5* | *medium* |
| Mimbella et al | 2016 | no | no | no | no | no | no | no | no | yes | yes | no | *2* | *high* |
| Parfene et al | 2016 | no | no | no | no | no | no | no | no | yes | yes | no | *2* | *high* |
| Arboleya et al | 2016 | no | yes | yes | yes | yes | no | no | yes | yes | yes | no | *7* | *medium* |
| Chen et al | 2016 | no | yes | no | no | yes | no | no | no | yes | yes | no | *4* | *medium* |
| Kar et al | 2017 | no | no | no | no | no | no | no | no | yes | yes | no | *2* | *high* |
| Lim et al | 2017 | no | no | no | no | no | no | no | no | yes | yes | no | *2* | *high* |
| Rong et al | 2017 | no | no | no | no | no | no | no | no | yes | yes | no | *2* | *high* |
| Tsutsui et al | 2017 | no | no | no | no | no | no | no | no | yes | yes | no | *2* | *high* |
| Foff et al | 2017 | no | yes | yes | yes | yes | no | no | no | yes | yes | no | *6* | *medium* |
| Jezequel et al | 2017 | no | yes | yes | yes | no | no | no | no | yes | yes | no | *5* | *medium* |
| Blackman et al | 2018 | no | no | no | no | no | no | no | no | yes | yes | no | *2* | *high* |
| Perero et al | 2018 | no | no | no | no | no | no | no | no | yes | yes | no | *2* | *high* |
| Scott et al | 2018 | yes | yes | yes | yes | yes | no | yes | yes | yes | yes | no | *9* | *low* |
| Dang-Vu et al | 2018 | no | no | no | no | no | no | no | no | yes | yes | no | *2* | *high* |
| Masopust et al | 2018 | no | no | no | no | no | no | no | no | yes | yes | no | 2 | *high* |
| Hanagasi et al | 2018 | no | no | no | no | no | no | no | no | yes | yes | no | 2 | high |
| Park et al | 2019 | no | no | no | no | no | no | no | no | yes | yes | no | 2 | high |
| Warren et al | 2020 | no | yes | yes | yes | yes | no | no | yes | yes | yes | no | 7 | medium |
| Laurikainen et al | 2020 | no | no | no | no | no | no | no | no | yes | yes | no | 2 | high |
| Endres et al | 2020 | no | no | no | no | no | no | no | no | yes | yes | no | 2 | high |
| Kaddu-Mulindwa | 2020 | no | no | no | no | no | no | no | no | yes | yes | no | 2 | high |
| Restrepo-Martínez et al | 2020 | no | no | no | no | no | no | no | no | yes | yes | no | 2 | high |
| Sinani et al | 2020 | no | no | no | no | no | no | no | no | yes | yes | no | 2 | high |
| Park et al | 2020 | no | no | no | no | no | no | no | no | yes | yes | no | 2 | high |

Supplemental Table 3: Characteristics of included patients

| **ID/Author/Year** | **Gender/ Age** | **Serum NMDA Abs/**  **Ig class/ Other abnormalities** | **CSF NMDA Abs/ Ig Class** | **Symptom Duration** | **Initial Diagnosis** | **Psychiatric Symptoms** | **Relevant Past medical History** | **CSF Pleocytosis/ OCB/ Protein/Other** | **MRI Abnormality/ Description** | **Time of EEG/ Concurrent treatment** | **EEG Dominant Rhythm/ Abnormalities** | **Follow up period/ Response to treatment** | **Immunotherapy** | **Other treatment** |
| --- | --- | --- | --- | --- | --- | --- | --- | --- | --- | --- | --- | --- | --- | --- |
| 1. Kung et al/2011 | M/24 | -/IgG/- | +/IgG | NA | NA | Psychosis | NA | +/-/-/- | -/- | >3 weeks/ Aripiprazole, haloperidol, lithium | NA/ Frontal slowing | NA/ full | IVMP, IVIg, RTX | Antipsychotics |
| 2. Tsutsui et al/2012 | F/26 | NA/NA/- | NA/NA | 1 year | Catatonic Schizophrenia | Psychosis, affective | Depression, BPAD, hypobulia, ovarian cyst | NA/NA/NA/NA | -/- | NA/ Antipsychotics*, ECT | NA/- | NA/ partial | - | Antipsychotics, ECT |
| 3. Hopkins et al/2013 | F/34 | +/IgG/- | NA/NA | 15 months | NA | Psychosis, affective, cognitive | Relapsing psychosis since 2007 | NA/+/-/- | -/- | >15 months/ Antipsychotics* | Delta/ Generalised slowing | 3 months/partial | Steroids, IVIg, PLEX, MMF | Antidepressants |
| 4. Kayser et al/2013 | F/18 | +/IgG/- | +/IgG | NA | NA | Psychosis, | NA | NA/NA/NA/NA | +/NOS | NA/NA | NA/- | 34 months/full | steroids, IVIg | NA |
| 5. Kayser et al/2013 | M/19 | NA/NA/- | +/IgG | NA | Demyelinating disease | Psychosis, affective, insomnia | NA | +/NA/NA/- | +/TLH, HI | >1 month/ Valproic acid | NA/- | 9 months/partial | Steroids, AZA | Valproic acid |
| 6. Kayser et al/2013 | F/46 | NA/NA/- | +/IgG | NA | NA | Psychosis, | NA | NA/NA/NA/NA | +/NOS | NA/NA | NA/ Abnormality NOS | 4 months/none | NA | NA |
| 7. Kayser et al/2013 | F/18 | +/IgG/- | +/IgG | NA | Relapse of anti-NMDAR encephalitis | Psychosis, affective | Anti-NMDAR encephalitis | NA/NA/NA/NA | -/- | NA/NA | NA/ Abnormality NOS | 13 months/full | IVIg | - |
| 8. Kayser et al/2013 | F/24 | -/NA/- | +/IgG | NA | Relapse of anti-NMDAR encephalitis | Psychosis, affective | Anti-NMDAR encephalitis | NA/NA/NA/NA | -/- | NA/NA | NA/ Abnormality NOS | 20 months/full | IVIg, Steroids, RTX | NA |
| 9. Kayser et al/2013 | F/24 | +/IgG/- | +/IgG | NA | Relapse of anti-NMDAR encephalitis | Psychosis, | Anti-NMDAR encephalitis | NA/NA/NA/NA | -/- | NA/NA | NA/ Abnormality NOS | 16 months/partial | PLEX, Steroids, IVIg AZA | NA |
| 10. Kayser et al/2013 | F/26 | +/IgG/- | +/IgG | NA | Relapse of anti-NMDAR encephalitis | Psychosis, affective | Anti-NMDAR encephalitis | NA/NA/NA/NA | +/NOS | NA/NA | NA/ Abnormality NOS | 28 months/partial | PLEX, IVIg, RTX | NA |
| 11. Kayser et al/2013 | F/28 | +/IgG/- | +/IgG | 33 months | HSV encephalitis. | Psychosis, affective | NA | NA/NA/NA/NA | +/HI | NA/NA | NA/ Abnormality NOS | 36 months/full | Steroids, IVIg, CTX, RTX | Valproic acid, quetiapine, chlorpromazine. |
| 12. Kayser et al/2013 | F/30 | NA/NA/- | +/IgG | NA | Relapse of anti-NMDAR encephalitis | Psychosis, | Anti-NMDAR encephalitis | NA/NA/NA/NA | -/- | NA/NA | NA/ Abnormality NOS | 25 months/ died | Steroids, RTX, CTX | NA |
| 13. Kayser et al/2013 | F/30 | -/NA/- | +/IgG | NA | Relapse of anti-NMDAR encephalitis | Psychosis, | Anti-NMDAR encephalitis | NA/NA/NA/NA | +/NOS | NA/NA | NA/ Abnormality NOS | 80 months/none | Steroids, IVIg, PLEX, CTX | NA |
| 14. Kayser et al/2013 | F/34 | -/-/- | +/IgG | NA | Relapse of anti-NMDAR encephalitis | Psychosis, | Anti-NMDAR encephalitis | NA/NA/NA/NA | -/- | NA/NA | NA/ Abnormality NOS | 22 months/partial | IVIg | NA |
| 15. Kayser et al/2013 | M/34 | -/-/- | +/IgG | NA | Relapse of anti-NMDAR encephalitis | Psychosis, affective | Anti-NMDAR encephalitis | NA/NA/NA/NA | +/NOS | NA/NA | NA/- | 44 months/partial | Steroids | NA |
| 16. Kayser et al/2013 | F/62 | NA/NA/- | +/IgG | NA | Relapse of anti-NMDAR encephalitis |  | Anti-NMDAR encephalitis | NA/NA/NA/NA | +/HI | NA/NA | NA/ Abnormality NOS | 20 months/none | Steroids, CTX, RTX | NA |
| 17. Leypoldt et al/2013 | M/24 | +/IgG/- | +/IgG | 18 days | HSV Encephalitis relapse | Psychosis, affective, cognitive | HSV Encephalitis | +/+/+/- | +/TLH, HI | NA/- | NA/- | 4 months/partial | Steroids | Acyclovir |
| 18. Steiner et al/2013 | M/24 | +/IgM/- | -/NA | NA | Paranoid SCZ | Psychosis, | NA | NA/NA/NA/NA | -/- | NA/- | Alpha/- | NA/NA | NA | NA |
| 19. Steiner et al/2013 | F/35 | +/IgM/- | -/NA | NA | Paranoid SCZ | Psychosis, | NA | NA/NA/NA/NA | -/- | NA/- | Alpha/ generalised slowing | NA/NA | NA | NA |
| 20. Steiner et al/2013 | M/22 | +/IgA/- | -/NA | NA | Paranoid SCZ | Psychosis, | NA | NA/NA/NA/NA | -/- | NA/- | Alpha/- | NA/NA | NA | NA |
| 21. Steiner et al/2013 | F/44 | +/IgA/- | -/NA | 3 years | Paranoid SCZ | Psychosis, | NA | NA/NA/NA/NA | +/ATY | NA/- | Alpha/ Generalised slowing | NA/NA | NA | NA |
| 22. Steiner et al/2013 | F/60 | +/IgA/- | -/NA | 10 years | Paranoid SCZ | Psychosis, | NA | NA/NA/NA/NA | -/- | NA/- | Alpha/- | NA/NA | NA | NA |
| 23. Steiner et al/2013 | M/45 | '+/IgA, IgM/- | -/NA | 13 years | Paranoid SCZ | Psychosis, | NA | NA/NA/NA/NA | -/- | NA/- | Alpha/- | NA/NA | NA | NA |
| 24. Steiner et al/2013 | M/38 | +/IgM/- | -/NA | 14 years | Paranoid SCZ | Psychosis, | NA | NA/NA/NA/NA | +/ATY | NA/- | Alpha/- | NA/NA | NA | NA |
| 25. Steiner et al/2013 | M/44 | +/IgG/- | -/NA | 19 years | Paranoid SCZ | Psychosis, | NA | -/+/NA/- | +/HI | NA/- | Alpha/- | NA/NA | NA | NA |
| 26. Steiner et al/2013 | M/58 | +/IgA/- | -/NA | 20 years | Paranoid SCZ | Psychosis, | NA | NA/NA/NA/NA | -/- | NA/- | Beta/- | NA/NA | NA | NA |
| 27. Steiner et al/2013 | M/50 | +/IgG/- | -/NA | 21 years | Paranoid SCZ | Psychosis, | NA | NA/NA/NA/NA | -/- | NA/- | Alpha/- | NA/NA | NA | NA |
| 28. Steiner et al/2013 | M/54 | +/IgA/- | -/NA | NA | Major Depression | Affective, | NA | NA/NA/NA/NA | +/ATY | NA/- | Alpha/- | NA/NA | NA | NA |
| 29. Steiner et al/2013 | M/44 | +/IgA/- | -/NA | NA | Major Depression | Affective, | NA | NA/NA/NA/NA | -/- | NA/- | Alpha/- | NA/NA | NA | NA |
| 30. Attalla et al/2014 | F/31 | NA/NA/- | +/NA | 1 week | NA | Psychosis, affective, cognitive | NA | +/NA/NA/- | -/- | 7-14 days after presentation/ Haloperidol, lorazepam | NA/ L temporal and R parasagittal slowing | 10 weeks/partial | Steroids, PLEX, IVIG | Salpingo-oopherectomy |
| 31. Kattepur et al/2014 | F/36 | +/NA/- | NA/NA | 5 weeks | NA | Psychosis, affective | NA | NA/NA/NA/NA | -/- | >5 weeks/ Antipsychotics* | NA/- | 1 month/partial | Steroids | Oophorectomy, antiepileptics |
| 32. Kuppuswamy et al/2014 | M/35 | +/NA/- | +/NA | NA | NA | Psychosis, affective | NA | NA/NA/NA/NA | -/- | NA/- | NA/- | 4 months/full | Steroids, PLEX, AZA | Quetiapine, lorazepam |
| 33. Grebenciucova et al/2015 | F/33 | NA/NA/- | +/NA | 4 weeks | NA | Psychosis, catatonia | NA | NA/NA/-/TPOAB | -/- | NA/NA | NA/- | NA/Full | Steroids, PLEX | Teratoma resection |
| 34. Heresco- Levy et al/2015 | F/67 | +/IgG, IgM/- | NA/NA | 40 years | SCZ | Psychosis, affective | Headache | NA/NA/NA/NA | +/HI | 40 years/ Sulpiride, citalopram, lorazepam, promethazine | NA/ Generalised slowing, EDB | NA/partial | NA | D serine |
| 35. Kelleher et al/2015 | F/55 | +/NA/- | +/NA | NA | First episode psychosis | Psychosis, | NA | NA/NA/NA/NA | -/- | NA/NA | NA/ Generalised slowing | NA/NA | NA | - |
| 36. Kelleher et al/2015 | M/u | +/NA/- | -/NA | NA | First episode psychosis | Psychosis, | NA | NA/NA/NA/NA | -/- | NA/NA | NA/- | NA/NA | - | Psychiatric treatment NOS |
| 37. Kelleher et al/2015 | M/u | +/NA/- | -/NA | NA | First episode psychosis | Psychosis, | NA | NA/NA/NA/NA | -/- | NA/NA | NA/- | NA/NA | - | Psychiatric treatment NOS |
| 38. Kelleher et al/2015 | M/u | +/NA/- | -/NA | NA | First episode psychosis | Psychosis, | NA | NA/NA/NA/NA | -/- | NA/NA | NA/- | NA/NA | - | Psychiatric treatment NOS |
| 39. Kruse et al/2015 | F/49 | +/IgG/- | -/- | 3 years | NA | Affective, | NA | -/-/-/- | -/- | NA/NA | NA/ L temporal slowing | 17 months/partial | NA | NA |
| 40. Lalanne et/2015 | F/31 | NA/NA/- | +/NA | NA | NA | Psychosis, affective | NA | NA/NA/NA/NA | +/LLH, HI | NA/ Mirtazapine*, clorazepate*, clomipramine*, olanzapine* | NA/ L temporal slowing | 2 years/full | Steroids, IVIg | Clomipramine, olanzapine |
| 41. Lu et al/2015 | F/36 | -/NA/  Anti-GAD 65 | +/IgG | 4 days | NA | Psychosis, | Ongoing pregnancy. Significant weight loss over 6 months | +/-/-/Anti GAD 65 | -/- | NA/NA | NA/- | 5 months/full | Oral and IV Steroids, PLEX | Mood stabilisers, methimazole |
| 42. Senda et al/2015 | F/31 | NA/NA/TPOAB, TgAb, TRAb | +/IgG | 2 years | NA | Psychosis, cognitive | NA | -/-/-/- | -/- | NA/ Antipsychotics | NA/- | NA/Full | IVMP, IVIg | Levothyroxine |
| 43. Yoshimura et al/2015 | F/47 | NA/NA/- | +/IgG | 4 days | NA | Affective, catatonia | Delirious mania, depression with catatonia, BPAD | -/-/-/- | -/- | NA/ Quetiapine | NA/ Generalised slowing | 14 months/partial | NA | Antipsychotics |
| 44. Yoshimura et al/2015 | F/48 | NA/NA/- | +/IgG | 2 months | NA | Psychosis, affective, catatonia, cognitive | Schizoaffective disorder, postpartum depression, mania, depression with catatonia | -/-/-/- | -/- | NA/ Quetiapine, lithium, lorazepam | NA/- | 7 months/partial | - | Antipsychotics |
| 45. Arboleya et al/2016 | M/22 | +/IgG/- | NA/NA | >18 months | BPAD, Mania | Psychosis, affective | HIV | NA/NA/NA/NA | +/ATY | 18 months/ Olanzapine*, clonazepam*, valproic acid* | Beta/- | 6 years/partial | - | Olanzapine, valproic acid, clonazepam |
| 46. Chen et al/2016 | F/55 | +/NA/- | +/NA | 2 days | NA | Affective, | NA | +/NA/+/- | -/- | NA/NA | Alpha/ Generalised slowing | 6 months/partial | Steroids, IVIg | NA |
| 47. McKeon et al/ 2016 | F/36 | +/NA/NA | NA/NA | 15 years | Psychosis | Psychosis, catatonia, cognitive impairment | Anorexia nervosa | NA/NA/NA/NA | -/- | NA/NA | NA/ Low amplitude throughout with prominent diffuse fast wave activity | 19 months/ partial | IVIg, mycophenolate | Antipsychotics, ECT |
| 48. McKeon et al/ 2016 | M/19 | +/NA/NA | +/NA | 6 years | Schizoaffective disorder | Psychosis, affective, cognitive impairment, insomnia | Thymic hyperplasia treated with thymectomy | NA/NA/NA/NA | -/- | >5 years/NA | NA/ Focal slowing - Frontal, R temporal | 14 months/ full | IVIg, RTX | Antipsychotics, ECT |
| 49. Mimbella et al/2016 | F/25 | NA/NA/- | +/NA | 3 months | NA | Psychosis, affective, insomnia | NA | NA/NA/NA/NA | -/- | > 3 weeks/ Divalproex*, Pramipexole* | NA/- | NA/partial | Steroids, PLEX | Hydroxyzine, divalproex, pramipexole, and levothyroxine |
| 50. Parfene et al/2016 | F/34 | +/NA/- | +/IgG | 5 weeks | Depression with psychotic features | Psychosis, affective, catatonia, cognitive | Encephalitis (possibly viral or autoimmune) | NA/NA/NA/NA | +/TLH, HI | NA/ Olanzapine, levetiracetam*, oxcarbazepine* | NA/ L temporal slowing | 3 weeks/partial | Steroids, IVIg, RTX | Antipsychotics, lorazepam |
| 51. Dang-Vu et al/2017 | M/23 | NA/NA/- | NA/NA | 10 days | Primary psychiatric/ Limbic Encephalitis | Psychosis, affective, catatonia, cognitive, insomnia | Diarrheal illness 10 days prior | +/-/-/- | +/LLH, HI, ATY | 1 day/ Olanzapine* | NA/ Generalised slowing, temporal focal slowing | NA/partial | Steroids, IVIG, Rituximab | Acicylovir |
| 52. Foff et al/2017 | F/35 | -/-/- | +/IgG | 4 days | NA | Insomnia | NA | NA/NA/+/- | -/- | 10 days/ Benzodiazepine, antiepileptics | NA/ | NA/partial | Steroids, IVIg, PLEX, RTX, CTX | Teratoma resection |
| 53. Jezequel et al/2017 | M/36 | +/IgG/- | -/- | 18 years | SCZ | Cognitive, dissociation | NA | -/-/+/CSA | -/- | NA/NA | NA/- | NA/partial | - | Antipsychotics |
| 54. Jezequel et al/2017 | M/44 | +/IgG/- | -/- | 22 years | SCZ | Cognitive, dissociation | Type 2 diabetes | -/-/+/- | -/- | NA/ Risperidone, cyamemazine, diazepam | Theta/ L temporo-occipital slowing, spike waves | NA/partial | - | Antipsychotics |
| 55. Jezequel et al/2017 | M/28 | +/IgG/- | -/- | 3 years | SCZ | Affective, cognitive | NA | -/-/+/CSA | -/- | NA/ Clozapine, levomepromazine, tropatepine | NA/ Bifrontal slowing, spike waves | NA/partial | - | Antipsychotics |
| 56. Jezequel et al/2017 | F/47 | +/IgG/- | -/- | 17 years | SCZ | Psychosis, cognitive, dissociation | Multiple ovarian cysts | -/+/+/- | -/- | NA/  Aripiprazole, tropatepine | NA/- | NA/partial | - | Antipsychotics |
| 57. Jezequel et al/2017 | F/21 | +/IgG/- | -/- | 5 years | SCZ | Affective, cognitive | Multinodular goitre, ovarian Sertoli Leydig tumour, multiple ovarian cysts, von Willebrand disease type 1 | -/-/+/CSA | -/- | NA/  Clozapine, tropatepine, propranolol | NA/ Generalised slowing | NA/partial | - | Antipsychotics |
| 58. Kar et al/2017 | M/21 | +/NA/- | -/NA | 2 days | NA | Psychosis, affective, catatonia, cognitive | Previous episode of sudden behavioural change | NA/NA/NA/NA | -/- | 7 weeks after discharge/Olanzapine, ECT | NA/- | 2 years/full | IVIg | Olanzapine |
| 59. Lim et al/2017 | M/65 | +/NA/- | +/NA | 1 week | NA | Psychosis, affective, cognitive | hypertension, hyperlipidaemia, diabetes | -/-/-/- | +/HI | NA/NA | NA/ | 6 months/partial | Steroids | - |
| 60. Rong et al/2017 | F/52 | NA/NA/TPOAB | +/IgG | 1 month | Severe depression with psychotic features | Psychosis, affective, cognitive | Cervical cancer, untreated depression | -/-/-/- | +/IS | NA/NA | NA/- | 2 months/full | IVMP, IVIg | Venlafaxine, lorazepam, olanzapine, ECT |
| 61. Tsutsui et al/2017 | F/60 | NA/NA/- | +/IgG | 2 weeks | NA | Psychosis, cognitive | Ovarian cyst | NA/NA/NA/NA | -/- | 2 weeks (After recovery) / Risperidone | NA/- | 15 months/full | - | Risperidone |
| 62. Blackman et al/2018 | M/37 | +/IgG/- | NA/NA | 4 days | HSV Encephalitis relapse | Psychosis, affective, cognitive, insomnia | HSV encephalitis | +/+/+/- | +/TLH, HI | >4 days/ Levetiracetam | Alpha/ R temporal slowing, EDB-like appearance | 4 months/full | - | Clonazepam, acyclovir |
| 63. Hanagasi et al/2018 | F/52 | +/IgG/NA | +/NA | 5 months | NA | Catatonia | BPAD depression with catatonia | NA/NA/+/NA | +/ATY | 5 months/ Quetiapine, pramipexole, levothyroxine | Theta/ Irregular theta waves | NA/ Partial | Steroids/ IVIg | NA |
| 64. Masopust et al/2018 | M/59 | +/IgG/NA | +/NA | 2 weeks | Psychosis | Psychosis, affective, cognitive impairment | Seminoma | NA/+/NA/NA | +/ATY | 7 weeks/ Levomepromazine, olanzapine | NA/ Slowing – right temporoparietal | 2.5 years/ partial | NA | Antipsychotics |
| 65. Perero et al/2018 | F/27 | +/NA/- | +/IgG | 2.5 years | Catatonia | Affective, catatonia, cognitive | NA | -/-/-/- | +/HI | 1.5 years/ Trazadone, Lorazepam, haloperidol, benztropine | Alpha with intermixed beta/- | 19 months/full | Steroids, IVIg, RTX | - |
| 66. Scott et al/2018 | M/33 | +/IgG/- | -/- | 2 days | BPAD | Psychosis, affective | NA | +/-/+/- | -/- | NA/ Risperidone*, quetiapine* | NA/- | 1 year 6 months/partial | IVMP, IVIg, AZA | Antipsychotics |
| 67. Endres et al/2019 | M/22 | +/IgG/- | -/NA | 3 days | Psychiatric- unspecified | Psychosis, affective, cognitive impairment, insomnia | NA | -/-/-/- | +/ Nonspecific bifrontal white matter lesions | NA/ Lorazepam | Beta/ Generalised slowing | 6 months/ full | Steroids | Lorazepam, diazepam |
| 68. Laurikainen et al/2019 | F/25 | +/IgG/- | +/IgG | 1 month | Mania with psychosis | Psychosis, affective, catatonia, cognitive impairment | NA | -/+/-/- | -/NA | >1 month/ Lithium | NA/- | 2.6 years/ full | Steroids, IVIg | ECT, antipsychotics, lithium |
| 69. Park et al/2019 | M/44 | +/NA/NA | +/NA | 5 days | NA | Psychosis, cognitive impairment | Anorexia | -/NA/+/NA | +/TLH, LLH | NA/NA | NA/ Right frontotemporal slowing | 2 years/ full | Steroids | Tumour resection |
| 70. Kaddu-Mulindwa et al/2020 | M/30 | +/IgG/HIV RNA | +/ IgG | 3 months | NA | Cognitive impairment | HIV | +/-/+/HIV RNA | -/NA | NA/NA | NA/- | 1 year/ partial | PLEX, RTX | NA |
| 71. Park et al/2020 | F/78 | NA/NA/NA | +/NA | 2 weeks | Parkinson’s disease | Catatonia | Anxiety, Hypertension | +/NA/+/- | +/HI | NA/ Lorazepam, bromocriptine, dantrolene | NA/ Generalised slowing | NA/ full | Steroids, IVIg, RTX | Lorazepam, bromocriptine, dantrolene |
| 72. Restrepo-Martinez et al/2020 | M/32 | NA/NA/NA | +/NA | 1 month | Delirious mania | Psychosis, affective, catatonia, cognitive impairment | NA | +/-/-/- | -/NA | 1 month/ Quetiapine, lorazepam | NA/ Generalised slowing | 1 month/ full | Steroids, IVIg, PLEX | Lorazepam, quetiapine, dexmedetomidine |
| 73. Sinani et al/2020 | F/27 | NA/NA/NA | +/ IgG | 5 months | NA | Affective, cognitive impairment | ADEM, NMOSD | +/+/+/  Anti-AQP4 IgG antibodies | +/ TLH, HI | 6 months/ steroids | Alpha/- | 4 years/ full | Steroids, IVIg, RTX | NA |
| 74. Warren et al/2020 | F/30 | +/IgG | +/NA | NA | Psychiatric - NOS | Psychosis, affective, insomnia | 1 patient had SCZ, 1 patient had EUPD | -/-/-/- | -/NA | 3 patients had concurrent medication (unspecified) | 3 patients had abnormal EEG with increased fast activity (medication related) | 319 days/ 5 patients improved with antipsychotics.  1 patient did not improve with antipsychotics or IVIg. | 1 patient received IVIg | Antipsychotics |
| 75. Warren et al/2020 | F/30 | +/IgG | -/NA | NA | Psychiatric - NOS | Psychosis, affective, insomnia |  | -/-/-/- | -/NA |  |  |  |  |  |
| 76. Warren et al/2020 | F/29 | +/IgG | -/NA | NA | Psychiatric - NOS | Affective, insomnia |  | -/-/-/- | -/NA |  |  |  |  |  |
| 77. Warren et al/2020 | F/29 | +/IgG | -/NA | NA | Psychiatric - NOS | Affective, insomnia |  | -/-/-/- | -/NA |  |  |  |  |  |
| 78. Warren et al/2020 | M/29 | +/IgG | -/NA | NA | Psychiatric - NOS | Affective |  | -/-/-/- | -/NA |  |  |  |  |  |
| 79. Warren et al/2020 | M/29 | +/IgG | -/NA | NA | Psychiatric - NOS | Affective |  | -/-/-/- | -/NA |  |  |  |  |  |

Time of EEG taken from onset unless specified, follow up period calculated from date of discharge. Warren et al 2020 cases reported as aggregate data. A = Abnormal (unspecified), ADEM = Acute disseminated encephalomyelitis, Anti GAD 65 = Anti-glutamic acid decarboxylase 65, ATY = Atrophy, AZA = Azathioprine, BPAD = Bipolar affective disorder, CSA (Elevated CSF/Serum albumin ratio), CTX= Cyclophosphamide, ECT= Electroconvulsive therapy, EDB = Extreme delta brush, EEG = Electroencephalogram, EUPD = Emotionally Unstable Personality Disorder, F= Female, HI = Hyperintensity (other) HSVE = Herpes simplex virus-1 encephalitis, Ig = Immunoglobulin, IVIg = IV Immunoglobulin therapy, L= Left, LE = Limbic Encephalitis, LLH = Limbic lobe hyperintensity, M = Male, MMF= mycophenolate mofetil, NA = Not available, NMOSD = Neuromyelitis optica spectrum disorder, NOS= Not otherwise specified, OCB = oligoclonal bands, PLEX = Plasma exchange, R= Right, SCZ= Schizophrenia, TLH = Temporal lobe hyperintensity, TgAb = anti-thyroglobulin antibody, TPOAB = anti-thyroid peroxidase antibodies, TRAb = thyrothropin receptor antibody. *indicates – ambiguous. Cingulate cortex considered to be part of limbic lobe.

Supplemental Section references

s1. Arboleya S, Clemente A, Deng S, Bedmar M, Salvador I, Herbera P, Cunill V, Vives-Bauza C, Haro J, Canellas F, Julia M (2016) Anti-NMDAR antibodies in new-onset psychosis. Positive results in an HIV-infected patient. Brain, Behavior, and Immunity 56 (pp 56-60), 2016 Date of Publication: 01 Aug 2016 56:56-60

2. Attalla S, Hemel D (2014) Woman, interrupted: An unusual cause of psychosis. Journal of General Internal Medicine Conference: 37th Annual Meeting of the Society of General Internal Medicine, SGIM 2014 San Diego, CA United States Conference Publication: (var pagings) 29 (pp S471), 2014 Date of Publication: April 2014 29:S471

3. Blackman G, Moran N, Brunnhuber F, Silber E, Symeon C, Mazumder A, Pollak T (2017) NMDA Receptor Autoimmunity In Mania Following HSV Encephalitis. Psychological Medicine

4. Chen Y, Xiang C, Huang Y, Zhang W, Wang G (2016) Clinical analysis of 9 cases of anti-NMDAR encephalitis. Medical Journal of Chinese People's Liberation Army 41 (9) (pp 767-770), 2016 Date of Publication: 2016

5. Dang-Vu G WA, Schmidt J (2020) A Rare Cause of New Onset Psychosis in a Young Male. In, Medical College of Wisconsin, Milwaukee, WI

6. Endres D, Rauer S, Kern W, Venhoff N, Maier SJ, Runge K, Süß P, Feige B, Nickel K, Heidt T, Domschke K, Egger K, Prüss H, Meyer PT, Tebartz van Elst L (2019) Psychiatric Presentation of Anti-NMDA Receptor Encephalitis. Frontiers in Neurology 10

7. Foff E, Taplinger D, Suski J, Lopes M, Quigg M (2017) EEG Findings May Serve as a Potential Biomarker for Anti-NMDA Receptor Encephalitis. Clinical EEG and Neuroscience 48 (1) (pp 48-53), 2017 Date of Publication: 2017 48:48-53

8. Grebenciucova E (2014) Catatonia: A Duel of Hashimoto’s Encephalopathy and Anti NMDA Receptor Encephalitis. In: Neurology. p P5.166

9. Hanagasi HA, Sezgin M, Bilgiç B, Seçkin M, Tufekcioglu Z, Gurvit HI, Emre M (2018) P4-085: CATATONIA AS CLINICAL PRESENTATION OF ANTI-N-METHYL-D-ASPARTATE (ANTI-NMDA) RECEPTOR ENCEPHALITIS. Alzheimer's & Dementia 14:P1467-P1468

10. Heresco-Levy U, Durrant AR, Ermilov M, Javitt DC, Miya K, Mori H (2015) Clinical and electrophysiological effects of D-serine in a schizophrenia patient positive for anti-N-methyl-D-aspartate receptor antibodies. Biological psychiatry 77:e27-29

11. Hopkins S, Moodley K, Chan D (2013) Autoimmune limbic encephalitis presenting as relapsing psychosis. BMJ Case Reports (no pagination), 2013 Article Number: 010461 Date of Publication: 30 Aug 2013:no pagination

12. Jezequel J, Johansson EM, Dupuis JP, Rogemond V, Grea H, Kellermayer B, Hamdani N, Le Guen E, Rabu C, Lepleux M, Spatola M, Mathias E, Bouchet D, Ramsey AJ, Yolken RH, Tamouza R, Dalmau J, Honnorat J, Leboyer M, Groc L (2017) Dynamic disorganization of synaptic NMDA receptors triggered by autoantibodies from psychotic patients. Nature communications 8:1791

13. Kaddu-Mulindwa D, Roth S, Klees-Rollmann A, Fassbender K, Fousse M (2020) Primary HIV infection presenting with Kaposi sarcoma and limbic encephalitis. Journal of NeuroVirology 26:292-296

14. Kar N, Kumar, M., Barreto, S. (2017) NMDAR antibody encephalitis and fluctuating catatonia. Progress in Neurology and Psychiatry 21:3

15. Kattepur AK, Patil D, Shankarappa A, Swamy S, Chandrashekar NS, Chandrashekar P, Prabhu S, Gopinath KS (2014) Anti-NMDAR limbic encephalitis--a clinical curiosity. World J Surg Oncol 12:256

16. Kayser MS, Titulaer MJ, Gresa-Arribas N, Dalmau J (2013) Frequency and characteristics of isolated psychiatric episodes in anti-N-methyl-d-aspartate receptor encephalitis. JAMA Neurol 70:1133-1139

17. Kelleher E, McNamara P, Fitzmaurice B, Walsh R, Langan Y, Whitty P, Gill M, Vincent A, Doherty C, Corvin A (2015) Prevalence rate of N-methyl-D-aspartate (NMDA) receptor antibodies in first episode psychosis. European Psychiatry Conference: 23rd European Congress of Psychiatry, EPA 2015 Vienna Austria Conference Publication: (var pagings) 30 (pp 1568), 2015 Date of Publication: 31 Mar 2015 30:1568

18. Kruse JL, Lapid MI, Lennon VA, Klein CJ, Toole OO, Pittock SJ, Strand EA, Frye MA, McKeon A (2015) Psychiatric Autoimmunity: N-Methyl-D-Aspartate Receptor IgG and Beyond. Psychosomatics 56:227-241

19. Kung DH, Qiu C, Kass JS (2011) Psychiatric manifestations of anti-NMDA receptor encephalitis in a man without tumor. Psychosomatics 52:82-85

20. Kuppuswamy PS, Takala CR, Sola CL (2014) Management of psychiatric symptoms in anti-NMDAR encephalitis: a case series, literature review and future directions. Gen Hosp Psychiatry 36:388-391

21. Lalanne L, Jantzi C, Gorse A, Zimmermann MA, Danion JM, Foucher J (2015) Melancholia Associated With Severe Cognitive Disorders as the Expression of Late-Onset Postpartum Anti-N-Methyl-d-Aspartic Acid Receptor Limbic Encephalitis. The Journal of neuropsychiatry and clinical neurosciences 27:e168-169

22. Laurikainen H, Isotupa I, Nyman M, Ilonen T, Nummelin T, Salokangas RKR, Hietala J (2019) Longitudinal brain morphology in anti-NMDA receptor encephalitis: a case report with controls. BMC psychiatry 19:145-145

23. Leypoldt F, Titulaer MJ, Aguilar E, Walther J, Bonstrup M, Havemeister S, Teegen B, Lutgehetmann M, Rosenkranz M, Magnus T, Dalmau J (2013) Herpes simplex virus-1 encephalitis can trigger anti-NMDA receptor encephalitis: case report. Neurology 81:1637-1639

24. Lim EW, Yip CW (2017) Anti-N-methyl-D-aspartate receptor encephalitis associated with hepatic neuroendocrine carcinoma: A case report. J Clin Neurosci 41:70-72

25. Lu J, Samson S, Kass J, Ram N (2015) Acute psychosis in a pregnant patient with Graves' hyperthyroidism and anti-NMDA receptor encephalitis. BMJ Case Rep 2015

26. Masopust J, Tvaroh A, Pavelek Z, Valis M (2018) Encephalitis with anti-NMDA receptor antibodies: paraneoplastic or non-paraneoplastic?. Neuro endocrinology letters 39:351-354

27. McKeon GL, Scott JG, Spooner DM, Ryan AE, Blum S, Gillis D, Langguth D, Robinson GA (2016) Cognitive and Social Functioning Deficits after Anti-N-Methyl-D-Aspartate Receptor Encephalitis: An Exploratory Case Series. J Int Neuropsychol Soc 22:828-838

28. Mimbella P, Verduzco-Gutierrez M, Irvine M, Podobinski T (2016) The rehabilitative management of a patient with anti-n-methyl-D-aspartate receptor encephalitis: A case report. PM and R Conference: 2016 Annual Assembly of the American Academy of Physical Medicine and Rehabilitation, AAPM and R 2016 United States 8 (9 Supplement) (pp S244-S245), 2016 Date of Publication: September 2016

29. Nicola Warren, M.B.B.S. ,, Andrew Swayne, M.B.B.S. ,, Dan Siskind, M.B.B.S., Ph.D. ,, Cullen O’Gorman, M.B.B.S., Ph.D. ,, Kerri Prain, B.Sci. ,, David Gillis, M.B.B.S. ,, Stefan Blum, Dr.Med., Ph.D. (2020) Serum and CSF Anti-NMDAR Antibody Testing in Psychiatry. The Journal of neuropsychiatry and clinical neurosciences 32:154-160

30. Parfene C, Lipira C, Gunning F, Gordon-Elliott J (2016) The neurocognitive profile of an anti-N-methyl-D-aspartate receptor encephalitis patient presenting with neuropsychiatric symptoms. Journal of Neuropsychiatry and Clinical Neurosciences 28 (3) (pp 255-256), 2016 Date of Publication: 2016 28:255-256

31. Park B-S, Son GM, Kim HS, Medina J, Cho JW (2019) Anti-N-methyl-d-aspartate receptor encephalitis in a patient with colon cancer. Clinical neurology and neurosurgery 177:114-116

32. Park DG, Kim TJ, Yoon JH (2020) Anti-NMDA receptor encephalitis presenting as catatonia associated with pheochromocytoma. Parkinsonism Relat Disord 72:62-64

33. Perero M, Reynolds J, Cahill J, Thompson B (2018) Two Sides of the NMDA Receptor Autoimmune Encephalitis Spectrum: the Tip of the Iceberg? Neurology:P5.381

34. Restrepo-Martinez M, Chacon-Gonzalez J, Bayliss L, Ramirez-Bermudez J, Fricchione GL, Espinola-Nadurille M (2020) Delirious Mania as a Neuropsychiatric Presentation in Patients With Anti-N-methyl-D-aspartate Receptor Encephalitis. Psychosomatics 61:64-69

35. Rong X, Xiong Z, Cao B, Chen J, Li M, Li Z (2017) Case report of anti-N-methyl-D-aspartate receptor encephalitis in a middle-aged woman with a long history of major depressive disorder. BMC Psychiatry 17:320

36. Scott JG, Gillis D, Ryan AE, Hargovan H, Gundarpi N, McKeon G, Hatherill S, Newman MP, Parry P, Prain K, Patterson S, Wong RCW, Wilson RJ, Blum S (2018) The prevalence and treatment outcomes of antineuronal antibody-positive patients admitted with first episode of psychosis. BJPsych Open 4:69-74

37. Senda M, Bessho K, Oshima E, Sakamoto S, Tanaka K, Tsutsui K, Kanbayashi T, Takaki M, Yoshimura B (2016) Anti-Inflammatory Therapy and Immunotherapy Were Partially Effective in a Patient With Anti-N-Methyl-D-Aspartate Receptor Antibodies and a Special Subgroup of Treatment-Resistant Schizophrenia. J Clin Psychopharmacol 36:92-93

38. Sinani AA, Maawali SA, Alshekaili J, Kindi MA, Ramadhani KA, Khabouri JA, Nadeem A, Salti AA (2020) Overlapping demyelinating syndrome (Neuromyelitis optica spectrum disorders NMOSD with anti-NMDA receptor encephalitis); A case report. Mult Scler Relat Disord 42:102153

39. Steiner J, Walter M, Glanz W, Sarnyai Z, Bernstein HG, Vielhaber S, Kastner A, Skalej M, Jordan W, Schiltz K, Klingbeil C, Wandinger KP, Bogerts B, Stoecker W (2013) Increased prevalence of diverse N-methyl-D-aspartate glutamate receptor antibodies in patients with an initial diagnosis of schizophrenia: specific relevance of IgG NR1a antibodies for distinction from N-methyl-D-aspartate glutamate receptor encephalitis. JAMA Psychiatry 70:271-278

40. Tsutsui K, Kanbayashi T, Takaki M, Omori Y, Imai Y, Nishino S, Tanaka K, Shimizu T (2017) N-Methyl-D-aspartate receptor antibody could be a cause of catatonic symptoms in psychiatric patients: case reports and methods for detection. Neuropsychiatr Dis Treat 13:339-345

41. Tsutsui K, Kanbayashi T, Tanaka K, Boku S, Ito W, Tokunaga J, Mori A, Hishikawa Y, Shimizu T, Nishino S (2012) Anti-NMDA-receptor antibody detected in encephalitis, schizophrenia, and narcolepsy with psychotic features. BMC Psychiatry 12:37

42. Yoshimura B, Yada Y, Horigome T, Kishi Y (2015) Anti-N-Methyl-D-Aspartate Receptor Encephalitis Presenting With Intermittent Catatonia. Psychosomatics 56:313-315
